# MYH9 binds to dNTPs via deoxyribose moiety and plays an important role in DNA synthesis

**DOI:** 10.18632/oncotarget.28219

**Published:** 2022-03-14

**Authors:** Pratima Nangia-Makker, Malathy P.V. Shekhar, Victor Hogan, Vitaly Balan, Avraham Raz

**Affiliations:** ^1^Barbara Ann Karmanos Cancer Institute, School of Medicine, Wayne State University, Detroit, MI 48201, USA; ^2^Department of Oncology, School of Medicine, Wayne State University, Detroit, MI 48201, USA; ^3^Department of Pathology, School of Medicine, Wayne State University, Detroit, MI 48201, USA; ^4^Guardant Health, Redwood City, CA 94063, USA

**Keywords:** myosin II, MYH9, dNTP, deoxyribose-5-phosphate, DNA synthesis

## Abstract

The accepted notion of dNTP transport following cytoplasmic biosynthesis is ‘facilitated diffusion’; however, whether this alone is sufficient for moving dNTPs for DNA synthesis remains an open question. The data presented here show that the MYH9 gene encoded heavy chain of non-muscle myosin IIA binds dNTPs potentially serving as a ‘reservoir’. Pull-down assays showed that MYH9 present in the cytoplasmic, mitochondrial and nuclear compartments bind to DNA and this interaction is inhibited by dNTPs and 2-deoxyribose-5-phosphate (dRP) suggesting that MYH9-DNA binding is mediated *via* pentose sugar recognition. Direct dNTP-MYH9 binding was demonstrated by ELISA and a novel PCR-based method, which showed that all dNTPs bind to MYH9 with varying efficiencies. Cellular thermal shift assays showed that MYH9 thermal stability is enhanced by dNTPs. MYH9 siRNA transfection or treatment with myosin II selective inhibitors ML7 or blebbistatin decreased cell proliferation compared to controls. EdU labeling and cell cycle analysis by flow cytometry confirmed MYH9 siRNA and myosin II inhibitors decreased progression to S-phase with accumulation of cells in G0/G1 phase. Taken together, our data suggest a novel role for MYH9 in dNTP binding and DNA synthesis.

## INTRODUCTION

Maintenance of genomic integrity requires tightly regulated preservation of dNTP levels and a delicate equilibrium between synthesis, consumption, and degradation processes. The availability of dNTPs in the nucleus and mitochondria is assumed to be a rate-limiting step in DNA synthesis and repair. The relationship between nuclear, mitochondrial, and cytoplasmic dNTP pools, and whether dNTP conveyance to the target for maintenance of equilibrium is unidirectional or bidirectional is unclear. It was suggested that the cytoplasmic and mitochondrial nucleotide pools are interconnected [1] as increases in the concentration of one dNTP causes imbalance in others; e.g., increases in cytoplasmic thymidine levels resulted in increases in mitochondrial dTTP and dGTP, and decreases in dCTP levels [2], indicating that diffusion *per se* cannot completely explain dNTP translocation. Furthermore, the reported evidence that the relationships among mitochondrial, cytoplasmic, and nuclear dNTP pools differ between normal and transformed cells [1] questions the notion of intracellular dNTP diffusion as the primary transport process. The presence of a myosin heavy chain ATPase-like polypeptide(s) as a nuclear envelope component was first reported in *Drosophila* [3] and later confirmed as part of the nuclear pore complex [4].

Myosins are an evolutionarily conserved superfamily of proteins. In humans, the 12 classes of myosins share common domains that interact with actin and hydrolyze ATP to generate energy for cell movement and maintenance of organelle/cell shape and cytokinesis [5]. Myosin II is the only family member that assembles into bipolar filaments with functional motor domains positioned at both ends of the filament in muscle and non-muscle cells. The filament formation is highly dynamic [6]. Myosin II exists as three isoforms, A, B, and C, which are hexameric enzymes composed of two heavy chains transcribed by MYH9, MYH10, and MYH14 genes, respectively, two essential light chains and two regulatory light chains. The heavy chain consists of a head, neck, and tail region: the head interacts with actin and binds ATP, the neck region binds to the light chains, and the tail region interacts with other proteins and is involved with dimerization [7]. Phosphorylation of the regulatory light chain on Ser19 and Thr18 is essential for its Mg-ATPase activity by controlling the conformation of the myosin head [8, 9]. Some members of myosin family also acts as cargo proteins [5].

In this paper, using cell free pull-down, mass spectrometry and direct binding analysis, we have identified that the myosin heavy chain (MYH9) component of myosin IIA selectively binds to dNTPs via the pentose sugar moiety. Consistent with inhibition of proliferation in MYH9 siRNA transfected cells, EdU labeling and cell cycle analysis of synchronized cells by flow cytometry demonstrated that inhibition of myosin II with blebbistatin or ML7 or depletion of MYH9 by MYH9 siRNA decreased progression to S-phase with accumulation of cells in G0/G1 phase. These data suggest an important new role for myosin II in dNTP binding and DNA synthesis.

## RESULTS

### The heavy chain of myosin IIA binds to DNA and is specifically eluted with 2-deoxyribose-5-phosphate (dRP)

**Figure 1:**
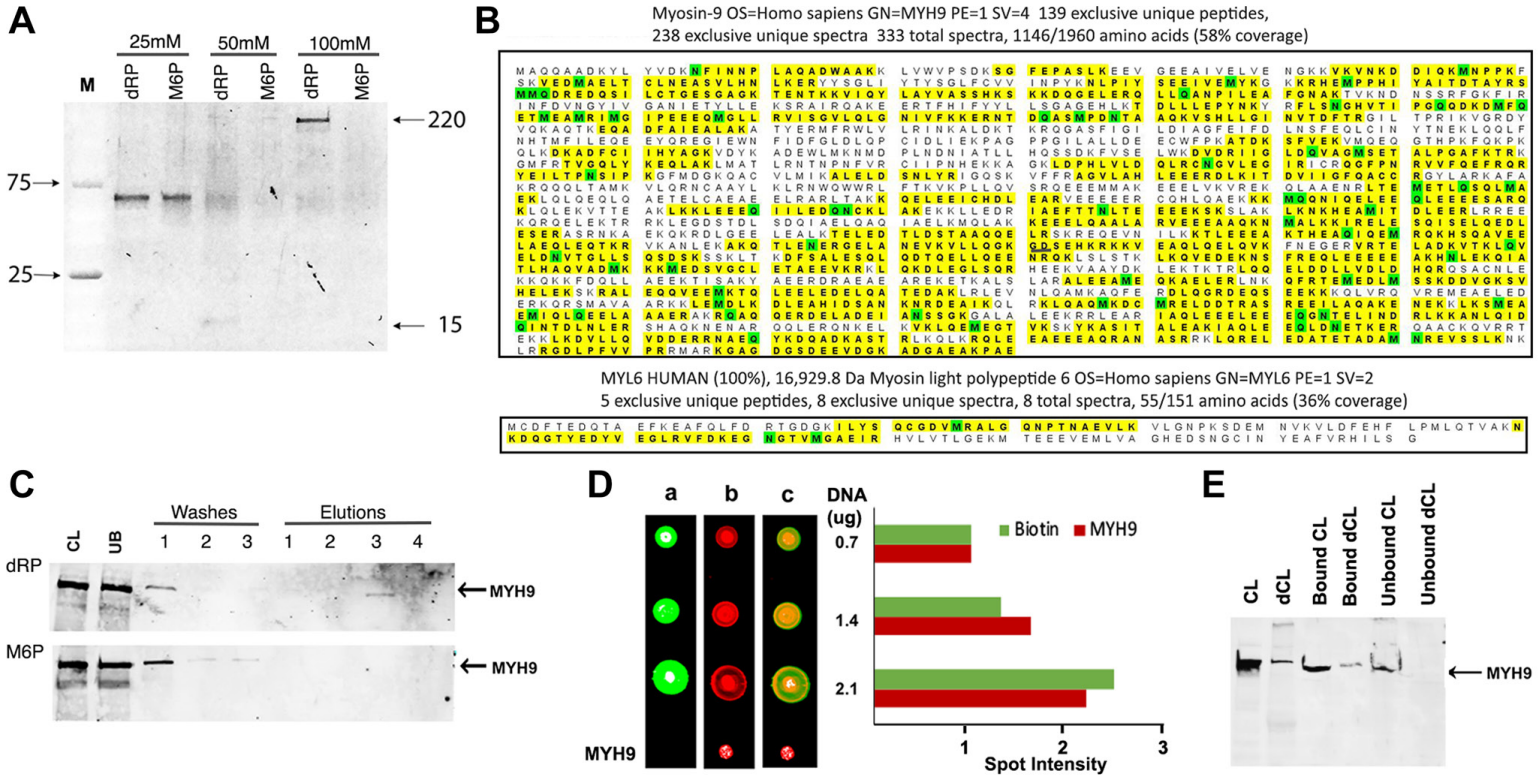
MYH9 binds to DNA via 2-deoxyribose-5-phosphate (dRP). (**A**) Elution of DNA binding proteins from MDA-MB-231 cells with 25, 50 or 100 mM dRP or mannose 6-phosphate (M6P). (**B**) Proteomic analysis revealed homology of eluted bands to Myosin heavy chain (MYH9: top) and light chain (MYL6: bottom). The yellow highlights indicate peptides with MYH9 and MYL6 sequence homologies identified in the sample. The green color indicates that at least one peptide with that residue highlighted was found to be modified. (**C**) Western blot analysis with anti-MYH9 antibodies: CL: cell lysate, UB: unbound protein, washes 1–3, and elutions (1–4) with 25, 50, 100 and 200 mM, respectively, of dRP (upper panel) or M6P (lower panel). (**D**) Binding of MYH9 to DNA immobilized on nitrocellulose membrane, detected by streptavidin conjugated IRDye 800 (green: lane a), anti-MYH9/anti-mouse IRDye 680 (red: lane b), and merged fields (lane c). Spot intensities were calculated by Image J software (**E**) 20 μg MYH9 immunodepleted MDA-MB-231 cell lysate (dCL) or control cell lysate (CL) was incubated with 1 μg biotinylated DNA and bound and unbound proteins were analyzed by western blotting with anti-MYH9 antibody.

To identify proteins with affinity for dNTPs, we used genomic DNA as a substratum as it is the ultimate destination of dNTPs. The initial experiments investigated if the proteins bound to DNA can be eluted with 2-deoxyribose-5-phosphate (dRP), the common constituent shared by all dNTPs. Biotinylated genomic DNA immobilized on streptavidin beads was incubated with whole cell lysates prepared from MDA-MB-231 human breast cancer cells. The bound proteins were eluted with increasing concentrations (25–100 mM) of dRP and resolved by gradient (4–20%) polyacrylamide gel electrophoresis (PAGE). D-mannose-6-phosphate (M6P), a phosphorylated sugar not present in DNA, RNA or in the nucleus but associated with well-defined signaling pathways, was used as a control. Staining of gels with Sypro Ruby (Invitrogen) revealed a band of ~200 kDa that was selectively eluted with 100 mM dRP and not with M6P (Figure 1A) and a smaller band of ~15 kDa that was eluted with 50 mM dRP (Figure 1A). A band of ~60 kDa was eluted both by dRP and M6P at 25 mM concentration (Figure 1A), which we did not pursue.

Proteomic analysis of the 200 and 15 kDa bands identified peptides with strongest homology to MYH9 (myosin heavy chain 9, molecular weight 220 kDa) (Figure 1B, upper panel) and MYL6 (myosin light chain 6, molecular weight 17 kDa) (Figure 1B, lower panel), respectively. To verify if the eluted 200 kDa protein is indeed MYH9, the washes and eluates were immunoblotted with anti-MYH9 antibody. The results confirmed that MYH9 eluted with 100 mM dRP (Figure 1C upper panel) but not with M6P (Figure 1C lower panel). These data indicate selective and strong binding of MYH9 to DNA via pentose sugar moiety as high concentration of dRP is needed to disrupt these interactions and release MYH9.

To further confirm MYH9 binding to DNA, increasing concentrations of biotinylated DNA were immobilized on nitrocellulose membrane and incubated with MDA-MB-231 cell lysates. MYH9-DNA binding was detected following sequential incubation with IRDye 800 conjugated streptavidin (Figure 1D, lane a), anti-MYH9 antibody and IRDye 680 conjugated secondary antibody (Figure 1D, lane b). Purified rabbit MYH9 was spotted as a positive control for antibody reactivity. Merged images (Figure 1D lane c) confirmed that MYH9 bound to the immobilized DNA, and density scans revealed proportional increases in the intensities of streptavidin and MYH9 signals with increasing concentrations of spotted DNA. MYH9 immuno-depleted cell lysates (dCL) showed decreased DNA binding (Figure 1E) compared to control cell lysates (CL) further supporting MYH9-DNA interaction.

### Subcellular localization of MYH9 and dRP selectivity for MYH9-DNA binding

**Figure 2:**
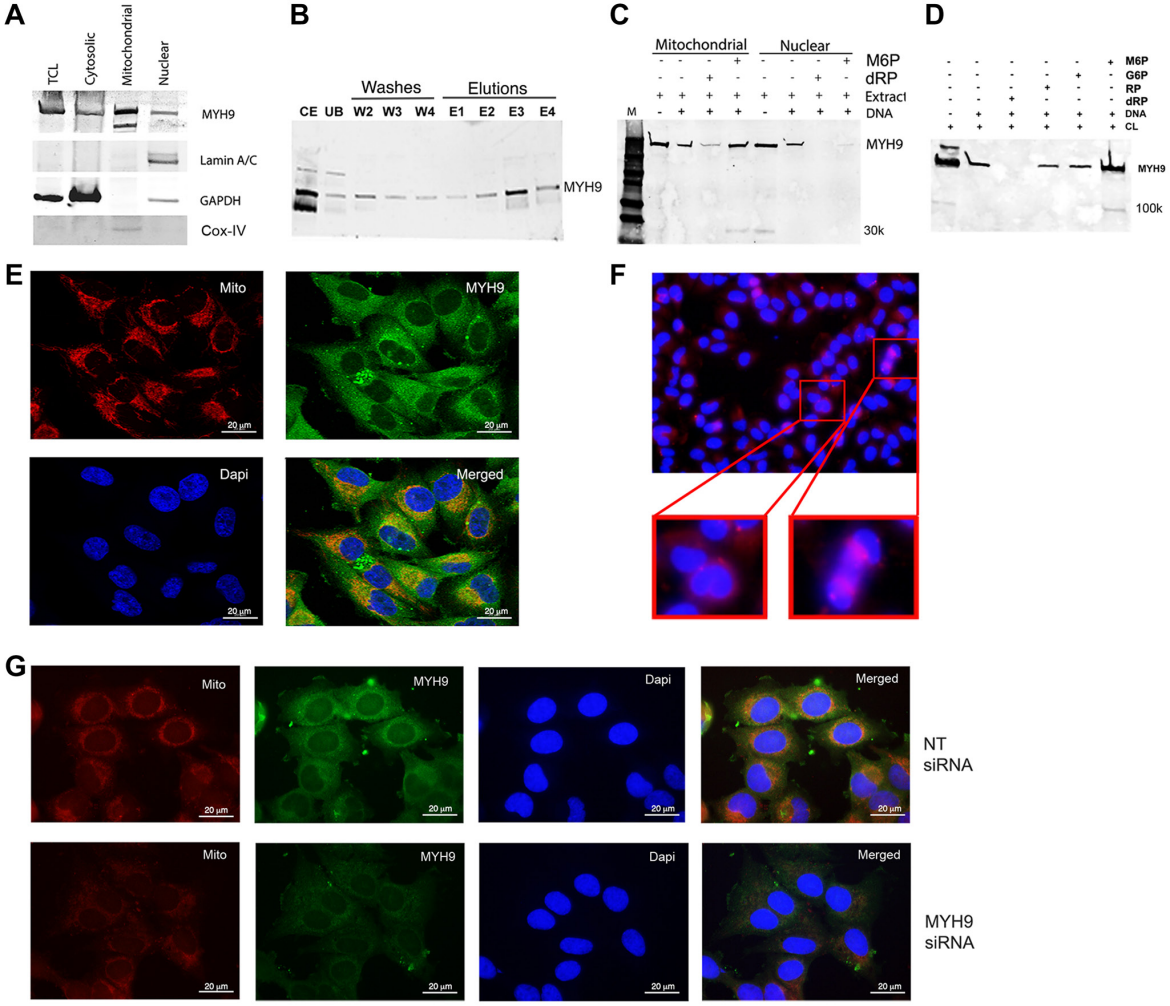
Intracellular localization of MYH9. (**A**) Western blot analysis of cell fractionation. Lamin A/C, GAPDH and Cox IV were used as nuclear, cytoplasmic and mitochondrial markers respectively. TCL: total cell lysate. (**B**) Binding of cytoplasmic fraction to DNA. Abbreviations: CE: cytoplasmic extract; UB: unbound proteins; W2-W4: washes 2–4; E1–E4: elutions with 25, 50, 100 or 200 mM dRP. (**C**) DNA-protein binding of mitochondrial or nuclear fractions in the presence of 100 mM M6P or dRP. (**D**) Biotinylated DNA (1 μg) was incubated with 50 μg cell lysate in the presence of 100 mM M6P, G6P, RP, and dRP and pulled down with streptavidin beads. The bound proteins were detected by anti-MYH9 antibody using SDS-PAGE. (**E**) Immunofluorescence staining of MDA-MB-231 cells with MitoTracker (red), MYH9 (green) and DAPI (blue) by confocal microscopy. (**F**) Immunofluorescence staining of MDA-MB-231 cells with anti-MYH9 antibody (red); inset shows dividing cells. (**G**) Mitotracker (red), MYH9 (green), Dapi (blue) staining following non-target (NT) (upper panel) and MYH9 (lower panel) siRNA transfection of MDA-MB-231 cells. The staining intensity of mitotracker was measured using adobe photoshop histogram and color range selection tools.

Western blot and immunofluorescence analyses were performed to determine the subcellular localization of MYH9 in MDA-MB-231 cells. MYH9 is present in the cytoplasmic, nuclear, and mitochondrial fractions (Figure 2A). Lamin A/C, GAPDH, and COX IV were used as markers for nuclear, cytoplasmic, and mitochondrial fractions, respectively. Binding assays showed that ~80, 40, and 50% of total input MYH9 from cytoplasmic, mitochondrial and nuclear subfractions respectively bound to DNA (Figure 2B–2D). The differences in binding capacity of MYH9 from the different subfractions could be attributed to the presence of various interacting molecular components in each compartment and/or differences in posttranslational modifications. Cytoplasmic MYH9 bound to DNA was eluted with 100 mM dRP (Figure 2B, lane E3) indicating strong affinity for DNA. Binding of mitochondria- and nucleus-derived MYH9 to DNA was also inhibited by dRP (Figure 2C) by approximately 60 and 80%, respectively, indicating strong pentose sugar-mediated interactions between DNA and MYH9 regardless of its subcellular origin. Surprisingly, binding of nuclear MYH9 to DNA was inhibited by M6P (Figure 2C). It is possible that other nuclear proteins may have facilitated this interaction. To determine the selectivity for sugar moiety, we compared the effects of dRP, M6P, glucose-6-phosphate (G6P), and ribose-5-phosphate (RP) on MYH9 binding to DNA. Binding assays were performed using whole cell lysates and biotinylated DNA in the presence of 100 mM dRP, RP, G6P or M6P. DNA/myosin-binding was inhibited by >85% by dRP and marginally by RP, whereas G6P and M6P had no inhibitory effect (Figure 2D). Smaller molecular weight bands (30 or 100 kDa, possibly cleavage products of myosin [7], were observed infrequently (Figure 2C, 2D), however, they showed no affinity for DNA.

Immunofluorescence staining showed robust MYH9 presence in the perinuclear region, but not in the nucleus (Figure 2E). Several investigators have failed to demonstrate MYH9 localization in the nuclear matrix by immunofluorescence. Currently, we cannot exclude the possibility that the MYH9 detected in the nuclear extract is derived from the perinuclear region (Figure 2E and 2G) or from cells undergoing cell division as intense MYH9 staining is observed in cells undergoing cytokinesis (Figure 2F).

The presence of myosin II in mitochondria and its role in maintenance of mitochondrial DNA integrity and fission has been previously suggested [10, 11]. Mitochondrial fission is a dynamic quality control process wherein unhealthy mitochondria are segregated and removed [10–12]. To define the association of MYH9 with mitochondria, we performed confocal microscopy analysis of cells stained with Mitotracker-Red, a mitochondrial potential-dependent dye, and anti-MYH9 antibody. Z-stack showed strong colocalization of MYH9 with active mitochondria. A co-localization coefficient of 0.994–0.998 was observed using Volocity software (Figure 2E). To determine whether MYH9 played a role in mitochondrial function, Mitotracker Red and anti-MYH9 reactivities were analyzed in cells transfected with non-target (NT) or MYH9 siRNAs. The staining intensities in NT and MYH9 siRNA transfected cells were compared using Adobe Photoshop software’s histogram tool. MYH9 depleted cells showed 9% active mitochondria compared to 28% in NT siRNA transfected cells (Figure 2G, lower and upper panels).

**Figure 3:**
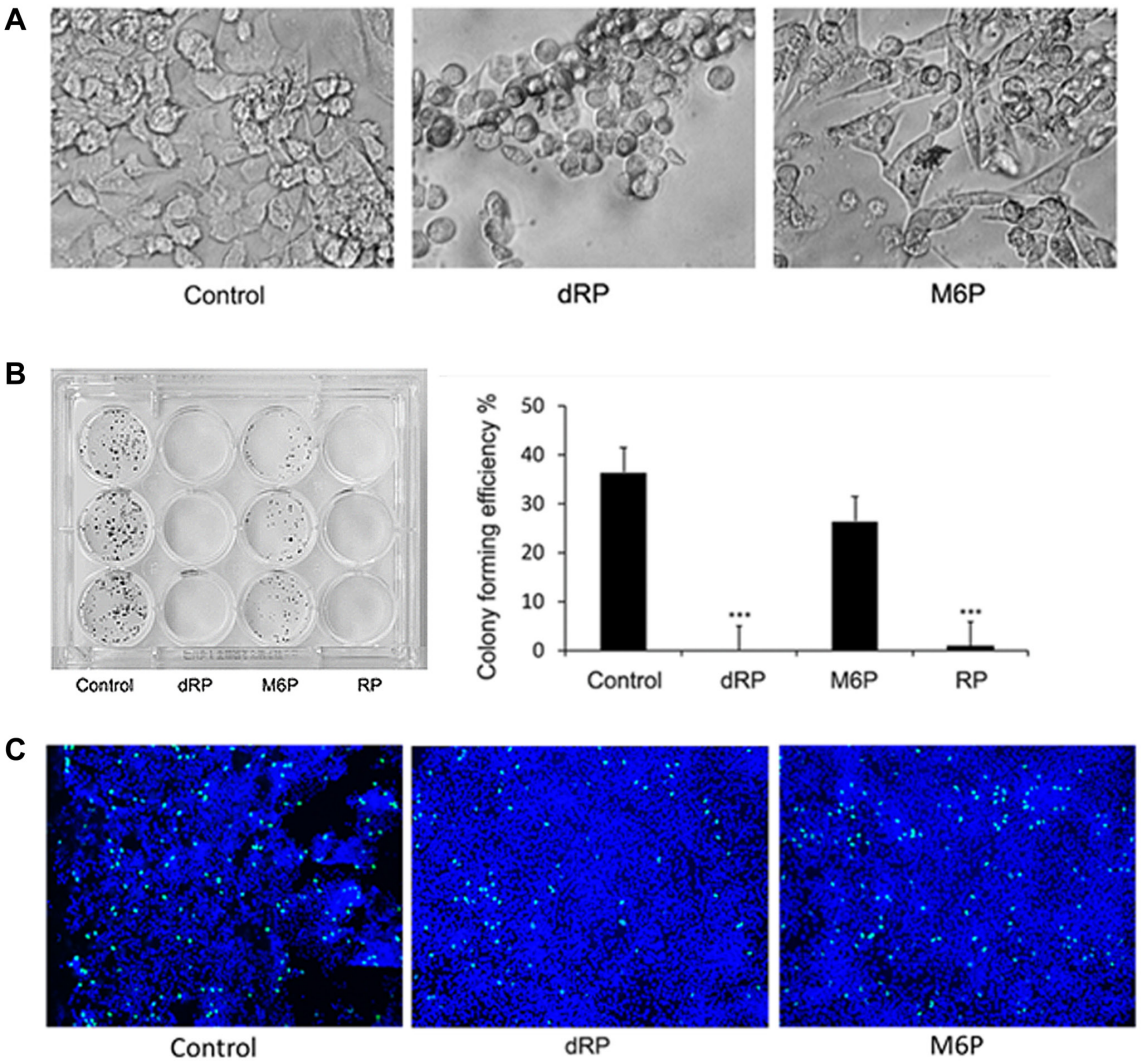
Effect of dRP on cell growth and morphology. (**A**) Cell morphology of 50 mM dRP and M6P treated MDA-MB-231cells after 24 hr. ×200. (**B**) Colony forming efficiency of MDA MB 231 cells. Cells were seeded with 6.25 mM dRP, M6P or RP and changed to normal medium after 48 hr. Right panel: graphical presentation of number of colonies. ^***^ *p* < 0.005 (**C**) EdU incorporation in cells treated with 6.25 mM dRP or M6P for 24 hr. Green color represents EdU incorporation and blue color represents Dapi counterstain ×100. Three fields from each slide were counted and % of replicating cells was calculated.

As dRP is able to inhibit MYH9-DNA binding, we proceeded to investigate its effect on cell morphology and growth. Treatment of MDA-MB-231 cells with dRP induced cell rounding and reduced colony forming potentials, while similar concentrations of M6P had no effect (Figure 3A, 3B). EdU (5-ethylyl-2′-deoxyuridine) incorporation assays were performed to determine the impact of dRP or M6P on cell replication potentials. While 3.8 and 3.3% of total cells were EdU-positive in control and M6P treated cells respectively, only 1.6% of dRP treated cells were EdU positive (Figure 3C). These data suggest that inhibition of DNA-myosin IIA interaction could potentially influence DNA replication.

### MYH9-DNA binding is inhibited by deoxyribonucleotides

**Figure 4:**
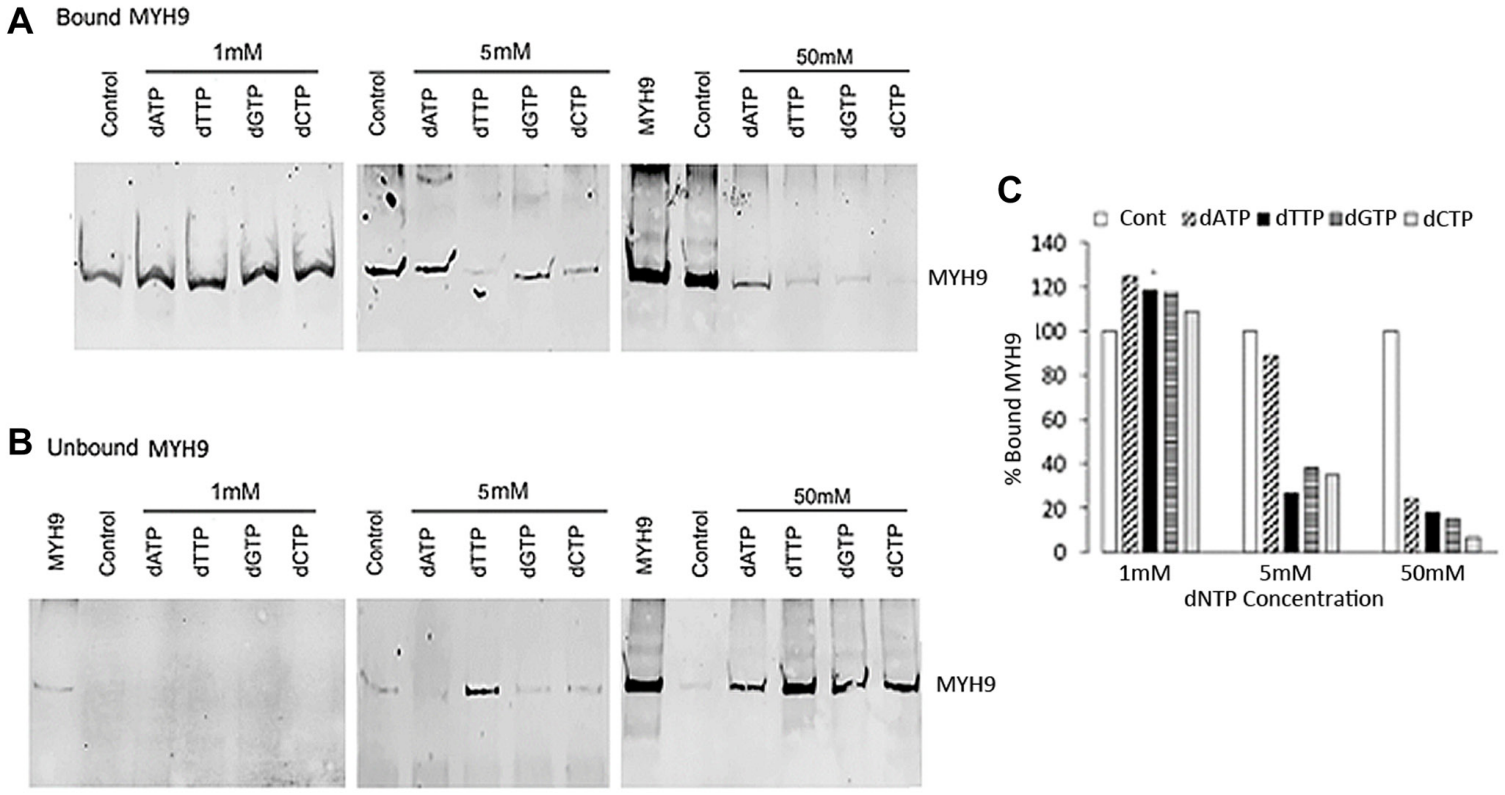
Inhibition of MYH9-DNA binding with dNTPs. (**A** and **B**) Purified MYH9-DNA binding in the absence (control) or presence of 1, 5 and 50 mM dATP, dTTP, dGTP or dCTP. (A): bound MYH9; (B): unbound MYH9 protein. Unbound MYH9 was detected following immunoprecipitation with anti-MYH9 antibody and pull-down with Protein G Sepharose. MYH9 was loaded as a marker in 1 and 50 mM unbound protein gels. (**C**) Graphical presentation of MYH9 bound to DNA in the presence of dNTPs as % of control.

To determine whether MYH9 binding to DNA is similarly inhibited by dNTPs as with dRP, we performed binding assays using genomic DNA and purified rabbit MYH9 in the presence of 1, 5, or 50 mM dATP, dGTP, dCTP or dTTP. While none of the dNTPs at 1 mM concentration were inhibitory, at 5 mM concentration all dNTPs other than dATP inhibited binding by >65%. Incubation with 50 mM dATP, dTTP, dGTP or dCTP inhibited MYH9-DNA binding by >75% with dCTP causing >90% inhibition (Figure 4A and 4C). Figure 4B show the levels of MYH9 in the corresponding unbound fractions. These data show that MYH9-DNA interaction is inhibited by dNTPs and that individual dNTPs bind to MYH9 with different affinities.

### *In vivo* MYH9-dNTP binding

**Figure 5:**
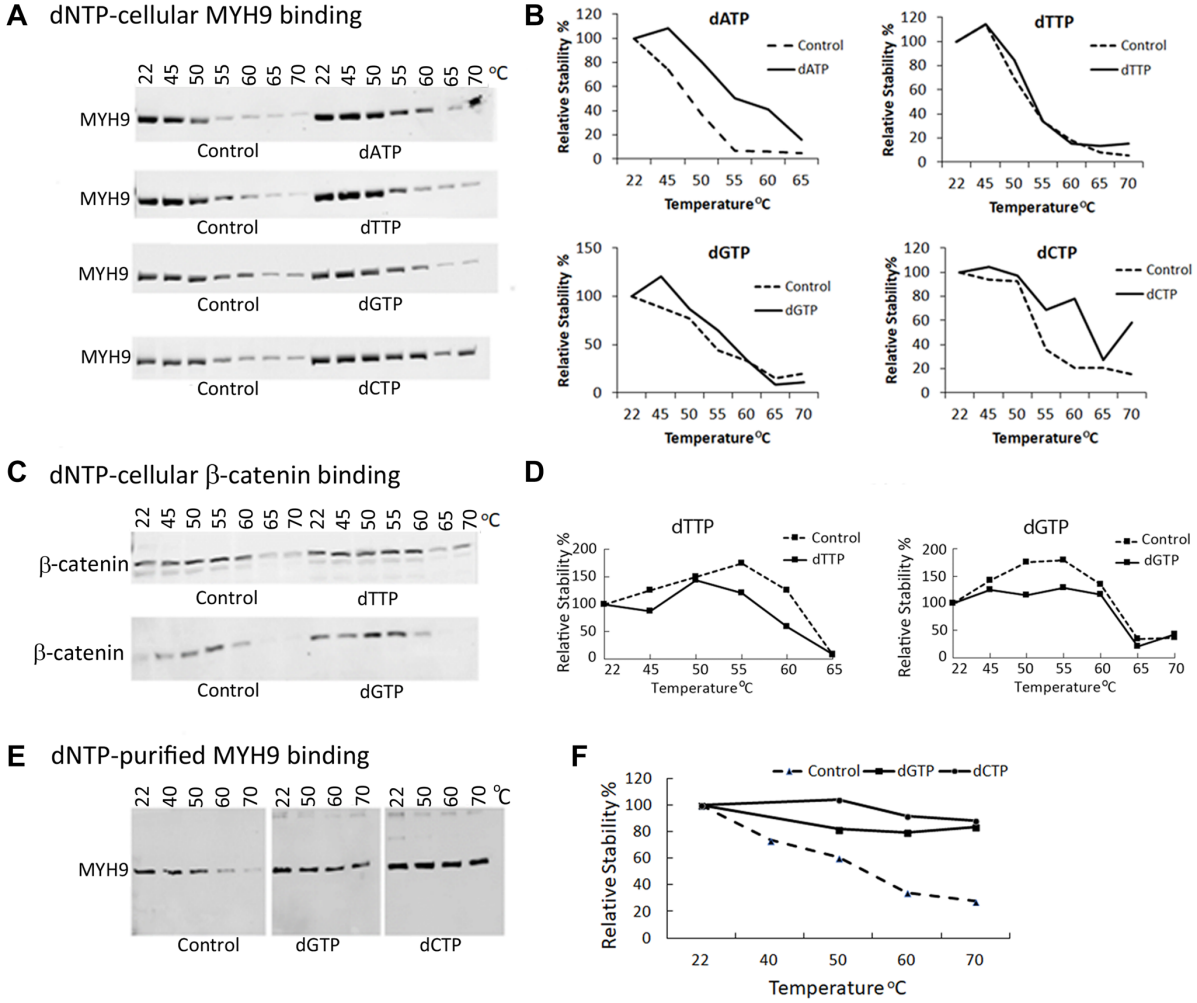
MYH9-dNTP binding analysis by cellular thermal shift assay (CETSA). (**A**–**D**) MDA-MB-231 whole cell lysates were preincubated with dNTPs at room temperature for 30 min prior to temperature escalation from 45°C to 70°C. MYH9 and β-catenin were detected by western blotting using anti-MYH9 antibody (A, B) and anti-β-catenin antibody (C, D). Purified MYH9 incubated with dNTPs was subjected to the indicated temperatures and analyzed by western blotting with anti-MYH9 antibody (**E**, **F**). (B, D, F) Graphical representation of A, C and E, respectively.

To determine *in vivo* dNTP-MYH9 binding, we performed cellular thermal shift assay (CETSA), which measures ligand-induced thermal stabilization of target proteins [13]. MDA-MB-231 cell lysates were incubated at room temperature for 30 min alone or in the presence of individual dNTPs and exposed to a temperature gradient ranging from 45–70^°^C. Unfolded or denatured proteins were removed by centrifugation and soluble thermostable proteins were detected by western blot analysis. The results in Figure 5A, 5B show increased thermostability of MYH9 in the presence of all dNTPs compared to control at temperatures 45°C to 60°C. Whereas at 55°C, untreated controls had ~30% thermostable MYH9, samples pretreated with dGTP, dCTP or dATP contained >50% thermostable MYH9 (Figure 5A, 5B). dTTP slightly stabilized MYH9 up to 50°C, but not at temperatures ≥55°C. These data further support that MYH9 binds to dNTPs with different affinities. Preincubation with dCTP produced greatest thermostability effects (up to 60°C), which is consistent with the results in Figure 4. To verify whether dNTP-induced thermostabilization effects were selective to MYH9, we analyzed the temperature and dNTP effects on β-catenin as it is also a large protein (90 kDa) and highly expressed like MYH9 in MDA-MB-231 cells. β-catenin protein degradation patterns were similar in control and dNTP pretreated samples (Figure 5C, 5D), confirming dNTP selectivity for MYH9 binding. To further validate the binding of dNTPs to MYH9, thermal shift assays were performed using purified MYH9 with or without preincubation with 0.1 mM dGTP or dCTP. As indicated in Figure 5E, 5F, compared to controls, both dGTP and dCTP pretreatments conferred thermostability to purified MYH9, confirming dNTP interaction with MYH9.

**Figure 6:**
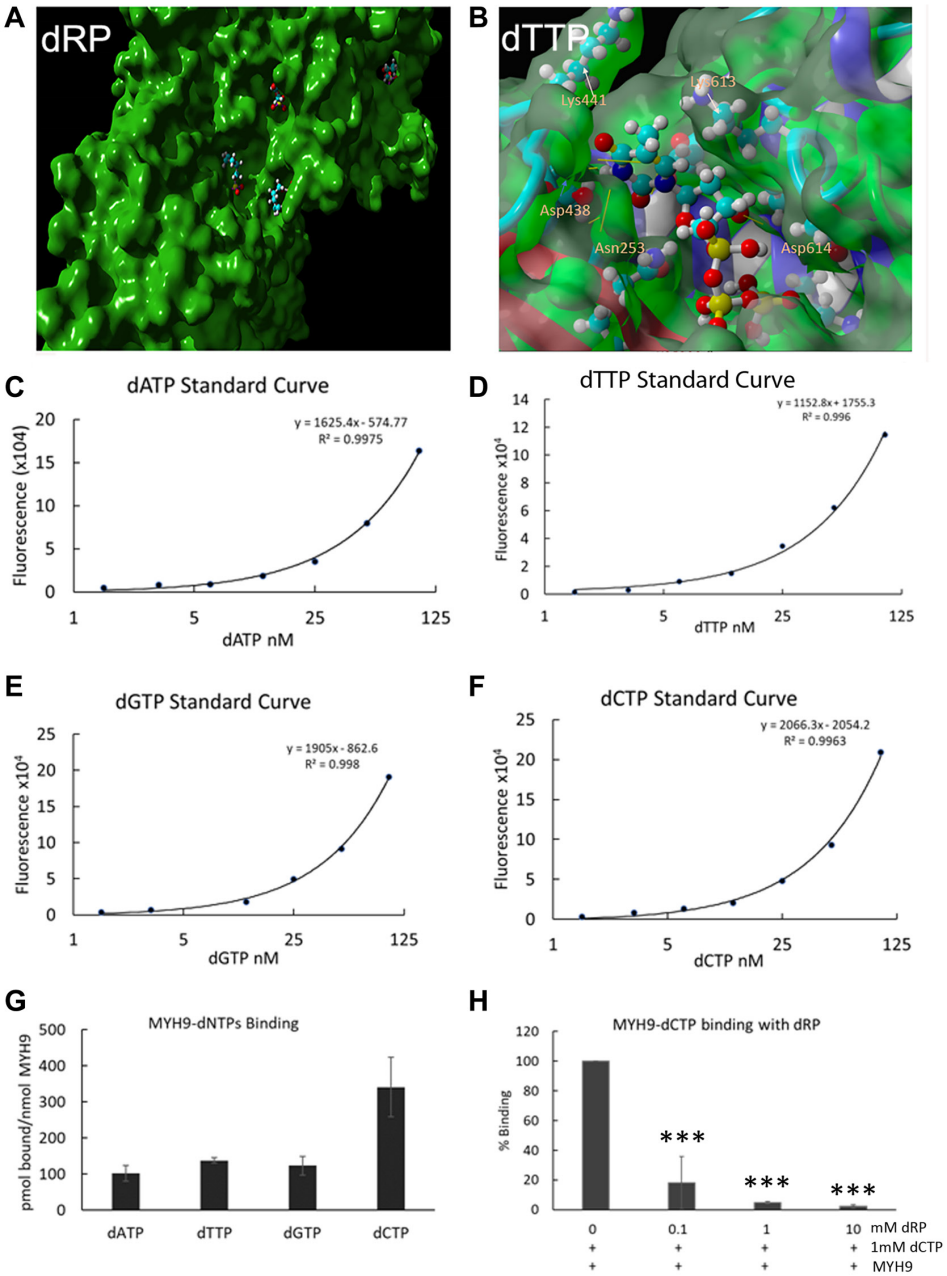
Direct binding of dNTPs to MYH9. (**A**, **B**) Docking analysis of dRP (A) and dTTP (B) with MYH9 head region (aa3-778). A magnified view of one pocket with interacting amino acids is shown in (B). (**C**–**F**) Standard curves of dATP, dTTP, dGTP and dCTP, respectively, using long synthetic oligonucleotide-based PCR method. (**G**) pmoles of individual dNTPs bound/nmole of MYH9. (**H**) Binding of dCTP to MYH9 in the presence of varying concentrations of dRP. Data represent average of three independent experiments. Bars represent ± standard deviation. ^***^ *p* > 0.005.

To get an insight into the potential interaction sites, molecular docking of dRP or individual dNTPs into MYH9 was performed and analyzed for binding energies and dissociation constant (Kd) in YASARA [14]. The dockings showed strong and stable binding with a positive YASARA score. The scores, Kd and contact receptor residues involved in MYH9 binding to dNTPs and dRP are shown in Supplementary Table 1. An example of dRP docking to the MYH9 C-terminus (amino acids 3–778) is shown in Figure 6A, which revealed several pockets for dRP binding. Similar docking analysis with individual dNTPs showed 14, 12, 15 and 11 distinct complex conformations for dATP, dCTP, dGTP, and dTTP, respectively, that differed by at least 5.0 A heavy ligand atom RMSD (root mean square deviation) after superimposing on the receptor (MYH9) (Supplementary Figure 1). A binding pocket with the interacting amino acids for dTTP is shown in Figure 6B. The binding energy of this interaction is 7.4680 kcal/mol with a Kd of 3.356475 μM. The residues involved in facilitating docking of individual dNTPs are shared in some docking pockets but not all. These data support the premise that MYH9 can bind all dNTPs with modest affinities.

To experimentally confirm dNTP-MYH9 binding affinity, we adopted the PCR-based method of dNTP analysis reported by Purhonen et al. [15] with slight modifications. Purified rabbit MYH9 (5 or 10 μg) was immobilized on an ELISA plate and its binding efficiency to the plate was determined by reaction with anti-MYH9 antibody and IRDye conjugated secondary antibody. Our results showed that about 90 and 82.5% of input MYH9, respectively, bound to the ELISA plate when 5 or 10 μg protein was plated. MYH9 coated plates were incubated with various dNTPs individually, washed, and the eluted MYH9-bound dNTPs were quantitated by PCR. The standard curves for individual dNTPs using the PCR-based method are shown in Figure 6C–6F), and the data in Figure 6G show that all dNTPs bind to MYH9. dCTP displayed the highest affinity with 341 pmol of dCTP bound/nmol of MYH9 compared to 101, 137 and 122 pmol of dATP, dTTP and dGTP, respectively, bound/nmol of MYH9. Although the binding constants of individual dNTPs could not be determined from this initial study, the data do confirm direct binding of all four dNTPs to MYH9. Addition of dRP caused marked reduction in dCTP binding to MYH9, further confirming that the binding is via the sugar moiety (Figure 6H). This study did not analyze whether dRP and dNTP compete for the same binding sites in MYH9, however, since the binding is via sugar moiety, we speculate there may be an overlap in the binding sites.

### Myosin II inhibition alters cytoplasmic dNTP pools and reduces DNA replication

**Figure 7:**
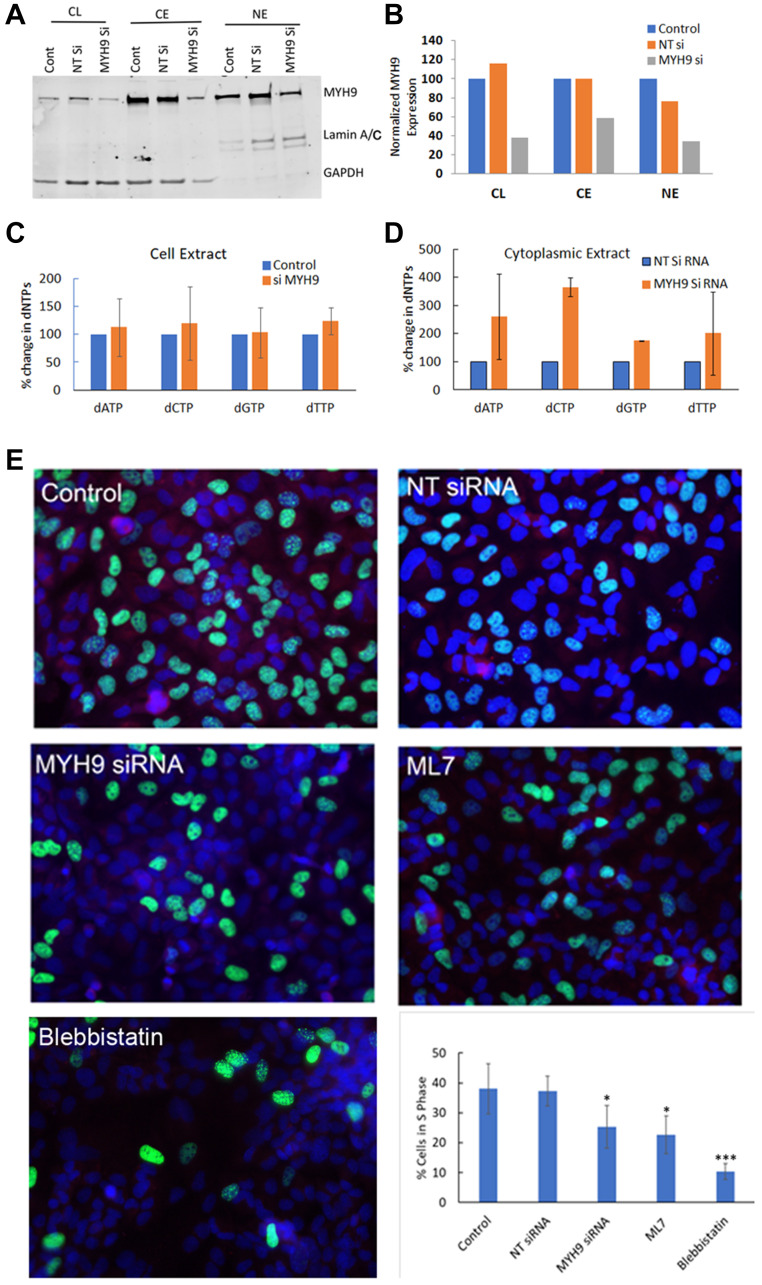
dNTP subcellular distribution and DNA synthesis by MYH9 depletion or inhibition. (**A**, **B**) Western blot analysis of MYH9 in total, cytoplasmic and nuclear fractions of non-target (NT) and MYH9 siRNA transfected MDA-MB-231 cells. Lamin A/C and GAPDH were used as nuclear and cytoplasmic markers, respectively. (**C**) Total dNTP pools in non-target control and MYH9 siRNA transfected MDA-MB-231 cells. (**D**) Cytoplasmic dNTP pools in nontarget control and MYH9 siRNA transfected MDA-MB-231 cells. ^*^ *p* < 0.01. Values are average of three independent experiments. (**E**) Edu Incorporation (green) and MYH9 staining (red) in cells treated with MYH9 siRNA and Myosin II functional inhibitors blebbistatin and ML7. ×400. Graph represents number of S phase cells as % of total cells. The numbers are average of five fields. *P* values were calculated for NT versus MYH9 siRNA, and control versus inhibitors. ^*^ *p* > 0.01, ^***^ *p* > 0.005. Bars represent ± standard deviation.

After having established that the heavy chain component of myosin IIA binds dNTPs, we next questioned the functional relevance of this binding. To determine whether MYH9 serves as a transporter of dNTP in the subcellular compartments, we analyzed the effect of MYH9 depletion on subcellular levels of MYH9 and dNTP. Compared to NT siRNA controls, MYH9 protein (Figure 7A, 7B) levels were decreased by ~40–50% in whole cell lysates, cytoplasmic and nuclear fractions in MYH9 siRNA transfected cells. The dNTP levels in whole cell lysates, cytoplasmic and nuclear fractions were analyzed by LC-MS/MS. While no significant differences in dNTP levels were observed in the total cell lysates of NT versus MYH9 siRNA transfected cells (Figure 7C), the levels of dATP, dCTP, dGTP and dTTP increased 161, 266, 75 and 101%, respectively, in the cytoplasmic fraction of MYH9 siRNA transfected cells as compared to NT siRNA transfected cells (Figure 7D). Nuclear dNTP levels could not be accurately and reliably measured potentially due to limitations in sensitivity of the method. Since MYH9 siRNA transfected cells showed consistent increases in cytoplasmic dNTP levels compared to NT siRNA transfected cells while maintaining similar dNTP levels in the total cell lysates, we speculate that the increases in cytoplasmic dNTP levels could potentially reflect decreases in nuclear dNTP levels in MYH9 siRNA transfected cells. These cells also displayed a reduced colony forming efficiency (Supplementary Figure 2). To determine whether MYH9 silencing or inhibition impact DNA synthesis, synchronized MDA-MB-231 cells were either transfected with NT or MYH9 siRNA, or treated with myosin II selective inhibitors blebbistatin or ML7 [16] and labeled with EdU. EdU positive cells were scored by fluorescence microscopy. Compared to NT siRNA transfected cells, MYH9 siRNA transfected cells showed a 12% reduction (*p* value 0.0025) in EdU positive cells (Figure 7E). Treatment with blebbistatin or ML7 resulted in much stronger inhibition of EdU incorporation with 28 and 16% decreases, respectively, as compared to controls (Figure 7E).

**Figure 8:**
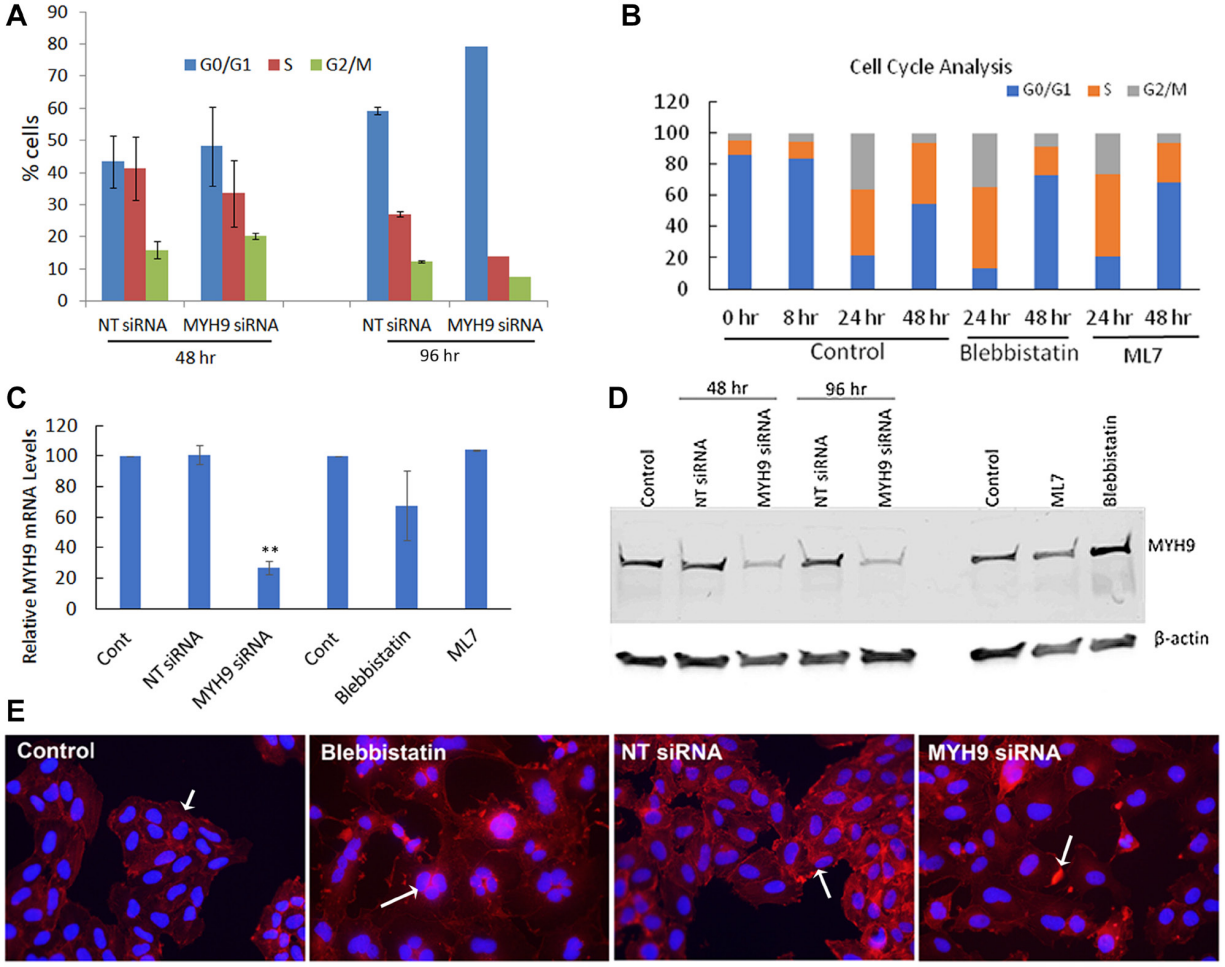
Cell cycle analysis. (**A**) Synchronized MDA-MB-231 cells were transfected with NT control and MYH9 siRNA 8 hr after release from serum starvation. Cell cycle analysis was performed after first and second rounds of transfection (48 hr and 96 hr respectively) by flow cytometry (**B**) Cell cycle analysis of MDA-MB-231 cells untreated or treated with blebbistatin and ML7 after 8 hr of release from serum starvation. Flow cytometric analysis of cell cycle was performed after 24 and 48 hr (**C**) MYH9 mRNA expression in siRNA transfected and inhibitor treated cells. GAPDH was used as a positive control. Values are average of 3 readings. *P* values were calculated for MYH9 versus NT siRNA ^**^ *p* > 0.002. (**D**) MYH9 protein expression in siRNA transfected, ML7 and blebbistatin treated MDA-MB-231 cells. β-actin was used as a positive control. (**E**) Immunofluorescence staining of F-actin (red) in cells transfected with siRNAs or treated with Blebbistatin. Arrows in control and NT siRNA control show F-actin filaments, multi nucleated cell in blebbistatin and actin dense spots in MYH9 siRNA treatment. Dapi (blue) was used as nuclear stain ×400.

To confirm whether MYH9 siRNA or Myosin II inhibitor induced decreases in EdU incorporation reflect inhibition of cell cycle progression, we performed cell cycle analysis of synchronized cells transfected with NT or MYH9 siRNAs. After first round of transfection, MYH9 siRNA transfected cells showed ~8% decrease in S phase cells and ~5 and 4% increases, respectively in G0/G1 and G2/M phase cells as compared to NT siRNA transfected controls (Figure 8A). Following a second round of transfection, MYH9 siRNA transfected cells showed 20% decrease in S-phase and 31% increase in G0/G1 cells as compared to NT controls (Figure 8A). In untreated control cells, an increase in S and G2/M phase was observed 24 hr after release from serum starvation (Figure 8B). At 48 hr, more cells passed through G2/M and entered G1/G0 phase, while the % of S phase cells remained the same. Compared to untreated controls, blebbistatin or ML7 treatment resulted in 33.4 and 28.2% decreases, respectively, in S-phase cells and 72 and 68% increases, respectively, in G0/G1 phase (Figure 8B). These results suggest a potentially important role for myosin IIA in DNA replication. The impact of myosin II inhibitors and the efficacy of MYH9 siRNA transfections on MYH9 gene expression and steady state protein levels were analyzed by quantitative real time RT-PCR and western blotting. Figure 8C, 8D shows a reduction by 75 and 70% in mRNA and protein levels respectively in MYH9 siRNA transfected cells compared to control and NT siRNA controls respectively, without any inhibition in cells treated with inhibitors.

As myosin is closely associated with actin filaments during cell division, we performed immunofluorescence staining of F-actin in MYH9 siRNA transfected and blebbistatin treated cells (Figure 8E). While in the untreated and NT control cells, strong actin filaments were observed, MYH9 siRNA and blebbistatin treated cells displayed lack of filamentous actin. Blebbistatin treatment also showed multinucleated cells, which could be due to their inability to pass through the cytokinesis successfully.

## DISCUSSION

The present study was undertaken to determine whether dNTP transport from the cytoplasm to the nucleus and mitochondria occurs by mechanisms besides diffusion. Using unbiased mass spectrometry analysis of DNA bound proteins eluted with deoxyribose-5-phosphate (dRP), we identified the heavy chain component MYH9 of myosin IIA as a major DNA binding protein, Utilizing recombinant MYH9 and subcellular cytoplasmic, mitochondrial and nuclear fractions, we further demonstrated that MYH9 binds to all four dNTPs via the deoxyribose sugar moiety, and that inhibition of myosin IIA or depletion of MYH9 alters cytoplasmic dNTP pools, decreases DNA synthesis and progression to S-phase. MYH9 is present in various cellular compartments and is involved in several activities [5, 7]. Our results show that MYH9 regardless of its cellular localization possesses the ability to interact with DNA, suggesting free movement of MYH9 pools within the cell. Immunofluorescence staining showed MYH9 localization in the mitochondria and perinuclear region but not in the nucleus although immunoblot analysis showed its presence in the nuclear subfractions. The presence of myosins I, II, V, VI, X, XVI, and XVIII has been described in the nucleus, however, they have been undetectable by immunofluorescence staining [reviewed by [17]]. The presence of MYH9 in the nucleus was demonstrated by ChIP assay [18], as well as by cell fraction analysis [19], but not by immunofluorescence using anti MYH9 antibodies. It is possible that nuclear myosin exhibits structural variability from its cytoplasmic counterpart, making it difficult for detection by routine staining/labeling methods [17]. Alternately, it is possible that nuclear MYH9 exists in a complex with other protein(s) potentially masking the epitopes. We also cannot exclude the possibility that MYH9 detected in the nuclear subfraction is derived from the perinuclear region. Presence of MYH9 at the cleavage furrow during cytokinesis and karyokinesis was reported earlier [20, 21].

Confocal microscopy and immunoblot analysis confirmed the presence of MYH9 in the mitochondria, and binding assays demonstrated dNTP binding by mitochondrial MYH9. Downregulation of MYH9 by siRNA transfection decreased the number of functional mitochondria, suggesting an important role for MYH9 in maintenance of healthy mitochondria.

Our data show that MYH9 binding to DNA is preferentially disrupted by dRP and not by RP, G6P or M6P, indicating that the deoxyribose sugar moiety specifies MYH9 binding to all four dNTPs. Cellular Thermal Shift Assays (CETSA) further confirmed that thermostability of MYH9 is enhanced by dNTP binding. Docking analysis of dRP and dNTPs to MYH9 revealed several pockets in MYH9 where dNTPs could bind. Quantitation of dATP, dCTP, dTTP and dGTP binding individually to recombinant MYH9 using a PCR based technique [15] showed that 341, 101, 137 and 122 pmoles of dCTP, dATP, dTTP and dGTP respectively bound/nmole MYH9. A stronger interaction of dCTP to MYH9 compared to rest of the dNTPs was also indicated by CETSA and MYH9-DNA binding analysis. Since MYH9-dCTP binding is inhibited by dRP, it further confirms overlap or competition for the binding pockets in MYH9 for the dNTPs. These binding sites are apparently different from the ATP binding site, which consists of the P-loop and switch I, situated at the boundary of N terminal and upper 50 kDa subdomains of the myosin head [22]. Overall, these results confirm binding of MYH9 to all dNTPs with strongest affinity for dCTP via the pentose sugar domain. The differences in their binding affinities may be due to differences in their structural properties, dCTP being the smallest and second most abundant dNTP in dividing cells, shows strong affinity. Although our results show a stronger affinity of MYH9 for dCTP, however, the availability of the nucleotides for DNA synthesis would depend on their release from myosin, which we have not analyzed in this study. Also, differential binding of dNTPs to MYH9 may be an inherent mechanism in the cells for keeping balance in the nucleotide pool.

Our LC-MS/MS data show that depletion of MYH9 by siRNA transfection increases cytoplasmic dNTPs without affecting the total dNTP pools. Due to experimental limitations, however, we could not determine whether this is accompanied by concomitant decreases in nuclear dNTP levels. Since myosin II is present as a filamentous structure in the cytoplasm with the ability to convert chemical energy into mechanical energy, we propose that it may be actively involved in translocation of nucleotides to the nucleus and mitochondria. Since MYH9 is strongly associated with the perinuclear and mitochondrial regions, we posit that MYH9 bound dNTPs may be enriched in these organelles.

Phosphorylation of myosin regulatory light chain at Ser19 and Thr18 is important for its unfolding, ATPase activity, and actin-binding [23]. The assembly of myosin II monomers into its filamentous structure is regulated by phosphorylation/dephosphorylation of the heavy chain at three Thr residues in the C-terminal region of the tail [24–26]. The myosin IIA inhibitors blebbistatin and ML7 used in our study inhibit Pi release from hydrolyzed ATP and inhibit myosin II phosphorylation, respectively [16, 27]. EdU labeling and cell cycle analysis showed that inhibition of myosin II with blebbistatin or ML7 reduced S-phase progression similarly to MYH9 siRNA supporting an important functional role for MYH9 in DNA synthesis. Cells treated with blebbistatin or MYH9 siRNA lacked well-formed actin filaments as seen in control or NT siRNA transfected cells. MYH9 depletion showed presence of concentrated actin focal areas, while blebbistatin treatment resulted in several multinucleated cells suggesting an important role of actin-myosin interactions in cell cycle progression.

**Figure 9:**
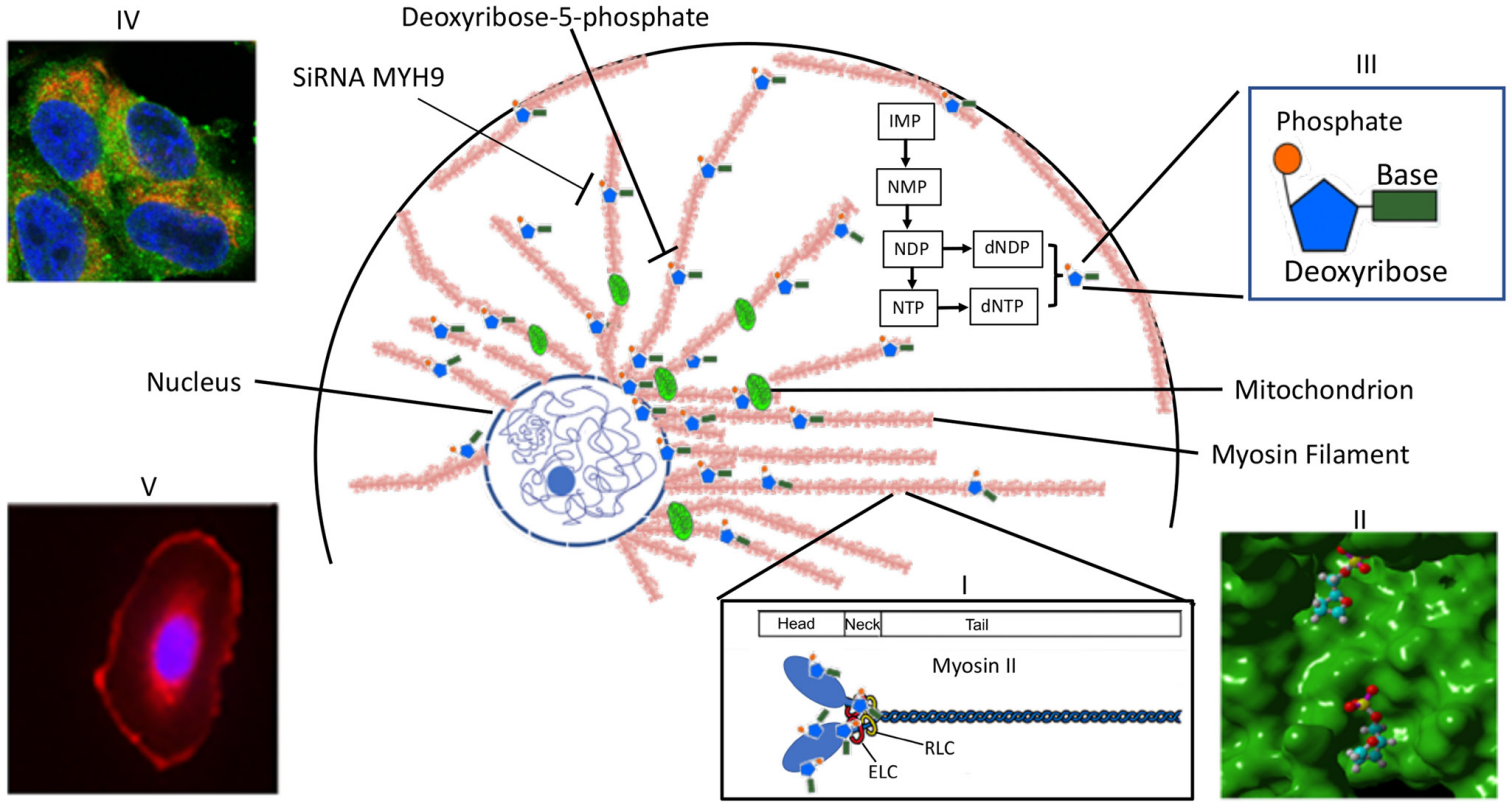
Scheme representing MYH9 role as a dNTP binding protein. Myosin II, which is polymerized into long thick filaments with MYH9 heads sticking out, binds to dNTPs *via* MYH9 heads. dNTPs bound to MYH9 concentrate in the perinuclear area and along the mitochondria to be available for DNA synthesis. Inset I: structure of myosin II: a pocket in myosin head with bound deoxyribose phosphate; III: a nucleotide phosphate; IV: Mitochondria showing colocalization of cytochrome c (red) and MYH9 (green); V: MYH9 peri-nuclear localization.

The results obtained above are summarized schematically in the model (Figure 9). The dNDPs and dNTPs are synthesized in the cytoplasm. MYH9 component of Myosin IIA recognizes and sequesters dNTP molecules via deoxyribose-5-phosphate moiety, and is concentrated around the nucleus and the mitochondria. The findings that the distribution of intracellular dNTP pools are different in transformed versus normal cells [1] and since increases in one nucleotide affects the distribution of the other three [2], along with our present findings strongly favor alternate dNTP transport mechanisms other than the current dNTP passive diffusion model. Since MYH9 is strongly expressed in the mitochondria and perinucleus, and it’s down regulation results in reduced DNA synthesis, we propose that it may serve as a reservoir of nucleotides for mitochondrial and nuclear DNA synthesis. Further studies are needed to monitor and visualize dNTP movement and their release from the myosin II filaments.

## MATERIALS AND METHODS

### Isolation, biotinylation and immobilization of genomic DNA on streptavidin beads

Genomic DNA was extracted from 80% confluent dishes of MDA-MB-231 cells using the Accuprep Genomic DNA extraction Kit by Bioneer Corp (Daejeon, Republic of Korea) according to the manufacturer’s instructions. DNA was digested with Sac II, precipitated, and suspended in TE buffer and biotinylated by thermal coupling with photoprobe biotin (Vector Laboratories, Burlingame, CA, USA) per manufacturer’s instructions. Unlabeled biotin was removed and DNA was precipitated and resuspended in TE.

Biotinylated DNA was pulled down by Dynabeads M-280 streptavidin using Dynabeads kilobaseBINDER kit (Invitrogen, Carlsbad, CA, USA) per manufacturer’s instructions. Briefly, the beads were incubated with DNA at room temperature with constant vertical shaking, washed and incubated with protein extract.

### DNA-protein binding and elution

Biotinylated DNA bound to Dynabeads was incubated overnight with MDA-MB-231 cell lysates in 20 mM Tris pH7.5, 150 mM NaCl, 1% Triton–X, and protease inhibitors with constant shaking. The beads were captured magnetically, washed and eluted with either 2-deoxyribose-5-phosphate (dRP) or mannose-6-phosphate (M6P) at 25, 50, 100, and 200 mM concentrations sequentially. The eluted samples were run on 4–20% gradient polyacrylamide gels, stained with Sypro Ruby, and the stained bands were submitted for mass spectrometry analysis at the Proteomics Core of Wayne State University.

### Mass spectrometry analysis

Gel pieces were processed as described previously [28]. The bands were then reduced with 5 mM DTT, alkylated with 15 mM iodoacetamide, and digested with sequencing-grade trypsin (Promega) in 40 mM Tris pH 8.0. Peptides were extracted using 50% ACN and 5% formic acid followed by separation by reversed-phase chromatography on Acclaim PepMap100 C18 column (Thermo Scientific). The separated peptides were ionized with the Nanospray Flex Ion Source (Thermo Scientific) and introduced into a Fusion mass spectrometer (Thermo Scientific).

Data were analyzed using Proteome Discoverer 2.1 (Thermo Scientific). The Uniprot_Hum_Compl_20170714 database was used, and a reverse decoy protein database was run to eliminate false discovery. The results were introduced into Scaffold 4.8 (Proteome Software) for distribution. Lowest protein identification odds were set at ≥99.0% with 2 unique peptides at ≥99.0% minimum peptide identification probability.

### Western blot analysis

Protein samples eluted with dRP or M6P from DNA bound fractions were subjected to SDS-PAGE on 4–20% gradient polyacrylamide gels (Bio-Rad laboratories, Hercules, CA, USA) and western blot analysis with a 1:1000 dilution of anti-MYH9 antibody (Sigma Aldrich, St. Louis, MO, USA). Following washes, the blots were reacted with 1:5000 dilutions of IRDye 680 or 800 conjugated corresponding secondary antibodies (Molecular Probes, Eugene, OR, USA) and scanned by Odyssey infrared imaging system (LI-COR Biosciences, Lincoln, NE, USA) to locate respective proteins. Subcellular analysis of MYH9 was similarly performed. Lamin A/C (Cell Signaling, Danvers, MA, USA; 1:1000 dilution), COX IV (Cell Signaling 1:1000 dilution) or GAPDH (Chemicon International, Temecula, CA, USA; 1:5000 dilution) were used as markers for nuclear, mitochondrial, or cytoplasmic fractions respectively.

### Binding of MYH9 to immobilized DNA

To confirm myosin binding to DNA, biotinylated genomic DNA (0.7, 1.4 and 2.1 μg) was immobilized on nitrocellulose membrane (MSI, Westboro, MA, USA) and UV crosslinked. As a positive control, 100 ng purified MYH9 (Sigma) was also spotted. The membrane was blocked with 1% casein and incubated overnight with MDA-MB-231 cell lysate (50 μg protein) at 4°C. DNA was detected by streptavidin conjugated IRDye 800, and the bound myosin was detected by incubation with anti-MYH9 (R&D laboratories, Antrim, UK) antibody and the corresponding anti-mouse IRDye 680. The membrane was scanned by the Odyssey infrared imaging system (LI-COR Biosciences, Lincoln, NE, USA).

### DNA-MYH9 binding in the presence of sugars and dNTPs

To determine if MYH9 binds to deoxyribose in DNA, binding assays were performed in the presence of various sugars. Biotinylated DNA (1 μg) was incubated overnight with 50 μg MDA-MB-231 cellular proteins in the presence of 100 mM dRP, ribose-5-phosphate (RP), D-glucose-6-phosphate (G6P), or M6P at 4°C with constant shaking for 15 hr. In some experiments, DNA and MYH9 binding was performed in the presence of dNTPs. Afterwards, the mixture was incubated with streptavidin Dynabeads, bound proteins were released by boiling with SDS sample buffer and detected by western blotting as described above. The unbound proteins (supernatant) were immunoprecipitated with anti-MYH9 antibody, pulled down with protein G sepharose and subjected to SDS-PAGE and western blot analysis.

### Isolation of cytoplasmic, nuclear, and mitochondrial fractions

The nuclear, cytoplasmic, and mitochondrial fractions were isolated using the Qproteome Mitochondria Isolation kit (Qiagen, Hilden, Germany) according to the manufacturer’s instructions. Equal amounts of subfractions were precipitated with acetone and subjected to SDS-PAGE and western blot analysis. The purity of cytoplasmic and nuclear fractions was confirmed by western blot analysis using GAPDH, Lamin A/C and COX IV antibodies as markers of cytoplasmic, nuclear, and mitochondrial fractions, respectively.

### Indirect immunofluorescence and confocal microscopy

The cells were seeded on an 8-chamber slide, fixed, permeabilized and processed for indirect immunofluorescence as described [29] using anti-MYH9 (Sigma 1:25) antibodies. Fluorescein isothiocyanate (FITC) or TRITC labeled anti-mouse or anti-rabbit IgGs (Sigma), at 1:100 dilutions were used as secondary antibodies. Mounting and counterstaining was performed with SlowFade Gold antifade reagent with 4′,6-Diamidino-2-Phenylindole Dihydrochloride (DAPI) (Invitrogen) and the cells were observed under an Olympus 1 × 71 microscope supporting a Hamamatsu 1394 ORCA-ERA video camera and stored using slidebook digital microscopy Software (Intelligent Imaging Innovations). Mitotracker (50 nm) (Molecular Probes, Eugene, OR, USA) was used to stain mitochondria in live cells (30 min incubation in 37°C incubator), followed by routine fixation and permeabilization steps as described above. Confocal microscopy was performed with a Zeiss LSM 780 microscope at Microscopy, Imaging and Cytometry Resource core of Karmanos Cancer Institute. Co-localization analysis was done using Volocity software (Perkin Elmer, Waltham, MA, USA).

### siRNA transfection

MDA-MB-231 cells were seeded in 6 well or 12 well plates to get approximately 50% confluence. siRNA transfections were performed with 20 pmole of Smart pool siRNAs specific to human MYH9 or nontarget (Dharmacon RNA Technologies, Lafeyette, CO, USA) using Lipofectamine RNAiMAX (Invitrogen) according to the manufacturer’s instructions. In some cases, cells were subjected to a second round of transfection. Cells were processed at 48 or 96 hr post-transfection. The sequences for On-Target plus human MYH9 Smart pool siRNAs were as follows:

GUAUCAAUGUGACCGAUUU, CAAAGGAGCCCUGGCGUUA, GGAGGAACGCCGAGCAGUA, CGAAGCGGGUGAAAGCAAA.

The sequences for non-target Smart pool siRNAs were as follows:

UGGUUUACAUGUCGACUAA, UGGUUUACAUGUUGUGUGA, UGGUUUACAUGUUUUCUGA, UGGUUUACAUGUUUUCCUA.

### Colony formation assay

MDA-MB-231 cells were seeded in the presence of 6.25 mM 2-deoxyribose-5-phosohate, D-mannose-6-phosphate or ribose-5-phosphate. Cultures were rinsed after 24 hr and placed in drug-free media. Colonies were allowed to form and were scored ~10 days later after staining with crystal violet. Colonies containing >50 cells were scored and colony forming efficiency was expressed relative to untreated controls.

### Cellular thermal shift assay (CETSA)

The effects of dNTPs on MYH9 thermostability was analyzed by cellular thermal shift assay (CETSA) as described by Molina et al. [13]. MDA-MB-231 cell lysates containing 50 μg protein were incubated with individual dNTPs (1 mM) for 30 min at room temperature. Control samples were incubated with buffer alone. Samples were aliquoted and heated for 3 min at 5°C intervals from 45°C–70°C. The denatured proteins were removed by centrifugation and the thermostable soluble proteins were subjected to SDS-PAGE and western blot analysis. Purified MYH9 (1 μg; Sigma Chemicals, St. Louis, MO, USA) was similarly incubated with 0.1 mM dGTP or dCTP and subjected to thermal denaturation as described above.

### LC-MS/MS analysis of dNTP levels

Cytoplasmic and nuclear subfractions were prepared from MDA-MB-231 cells transfected with NT or MYH9 siRNAs, and dNTP levels were analyzed by Liquid Chromatography with tandem mass spectrometry (LC-MS/MS) using AB SCIEX QTRAP 6500 system (Foster City, CA, USA) at the Pharmacology Core of Karmanos Cancer Institute. Data were acquired and analyzed using Analyst 1.6 and MultiQuant 3.0 software as described [30].

### PCR-based dNTP analysis

5 or 10 μg purified rabbit MYH9 (Sigma) was coated on MaxiSorp ELISA plates (Thermo Fisher, Waltham, MA, USA), and incubated overnight at 4°C with individual dNTPs (1 mM). Unbound dNTPs were removed by washing and MYH9-bound dNTPs were eluted with 1 mM glycine, pH 2.7 followed by immediate neutralization with 100 mM Tris HCl, pH 8.5. MYH9-bound dNTPs were assayed by PCR as described by Purhonen et al. [15]. Briefly, single strand DNA templates of 197 nucleotides specific for each of the four dNTPs were synthesized (Integrated DNA Technology). Each template consisted of a 3’primer binding site followed by a single dNTP detection site. Rest of the template did not contain the dNTP to be detected. Accumulating PCR products were detected with EvaGreen dye bound to double stranded DNA. PCR reaction mixture consisted of 0.275 μM primer, 0.25 μM template, 50 μM non-limiting dNTPs (dNTP mix without the dNTP to be detected), 1× Eva Green and 20 U/ml Q5 polymerase in a 20 μl reaction volume. PCR was performed using StepOnePlus Real-Time PCR System (Applied Biosystems) under following conditions: Step 1: 10 sec at 95°C, Step 2: 1 sec at 75°C, Step 3: read baseline fluorescence, Step 4: 1 sec at 66^°^C, Step 5: read fluorescence, Step 6: 5 min at 66°C. Repeat step 4–6 for 10 cycles, Step 7: 5sec at 75°C, Step 8: read end point fluorescence. The sequences for the DNA templates and primers are provided in Supplementary Table 2.

To determine the amount of MYH9 immobilized on the ELISA plate, the unbound and initial input samples were separated by PAGE and subjected to western blot analysis. The relative band intensities of input and unbound proteins were analyzed to determine bound MYH9.

### 5-ethynyl-2′-deoxyuridine (EdU) incorporation

Incorporation of EdU, a thymidine analog, during DNA synthesis was measured using Click-iT Plus EdU Imaging Kit (Molecular Probes, Eugene, OR, USA). Briefly, MDA-MB-231 cells seeded in 4-chamber slides were incubated with 10 μM EdU for 2 h, fixed with 3.7% formaldehyde and permeabilized with 0.5% Triton X-100. EdU incorporated into DNA was detected with Click-iT reagent as recommended by the manufacturer. Slides were counterstained with DAPI and images were collected using Olympus 1 × 71 microscope supporting a Hamamatsu 1394 ORCA-ERA video camera and stored using slidebook digital microscopy Software.

### Docking of MYH9 and 2-deoxyribose-5-phosphate or dNTPs

Homology model of Myosin-9 of Homo sapiens and Molecular docking of dRP to human MYH9 was performed using SWISSDock and SWISS-Model, respectively [31, 32]. The SWISS-Model template library (SMTL version 2018-07-22, PDB release 2018-07-13) was searched with BLAST [33] and HHBlits [34] for structures matching the target sequence and templates with the highest target-template alignment were selected for model building using ProMod3 or ProMod-II. Docking of dNTPs on human MYH9 was performed using VINA [35] and default parameters and the molecular graphics were created with YASARA [36].

### Cell cycle analysis

MDA-MB-435 cells were synchronized by serum starvation for 96 h, and cells were transfected with NT or MYH9 siRNAs, or treated with 37.5 μM blebbistatin or 25 μM ML7, 8 hr after release into complete media. Control untreated and inhibitor treated cells were collected after 24, 48 or 72 hr. siRNA transfected cells were collected after 48 h and at 96 h after a second round of transfection. Cells were fixed with 70% ethanol, incubated with DNase free RNase A and propidium iodide, quantitated by BD LSR II SORP flow cytometer (BD Biosciences, San Jose, CA, USA) at the Microscopy, Imaging, and Cytometry Resources Core of Karmanos Cancer Institute and analyzed using using ModFit LT v.5.0 (Verity Software House, Topsham, ME, USA).

## SUPPLEMENTARY MATERIALS





## Abbreviations

MYH9: myosin heavy chain 9
dRP: 2-deoxyribose-5-phosphate
R6P: ribose-6-phosphate
M6P: mannose-6-phosphate
G6P: glucose-6-phosphate
dATP: deoxyadenosine triphosphate
dTTP: deoxythymidine triphosphate
dGTP: deoxyguanosine trihosphate
dCTP: deoxycytidine triphosphate
EdU: 5-ethynyl-2′-deoxyuridine
CETSA: cellular thermal shift assay
DAPI: 4′,6-Diamidino-2-Phenylindole Dihydrochloride
