# Supplementary material for: MYH9 binds to dNTPs via deoxyribose moiety and plays an important role in DNA synthesis
Source: Oncotarget. 2022 Mar 14;13:534–50. doi: 10.18632/oncotarget.28219 (PMC8923078; doi:10.18632/oncotarget.28219)
Supplement: Supplementary file 1 [file oncotarget-13-28219-s001.pdf]

MYH9 binds to dNTPs via deoxyribose moiety and plays an important role in DNA synthesis

SUPPLEMENTARY MATERIALS

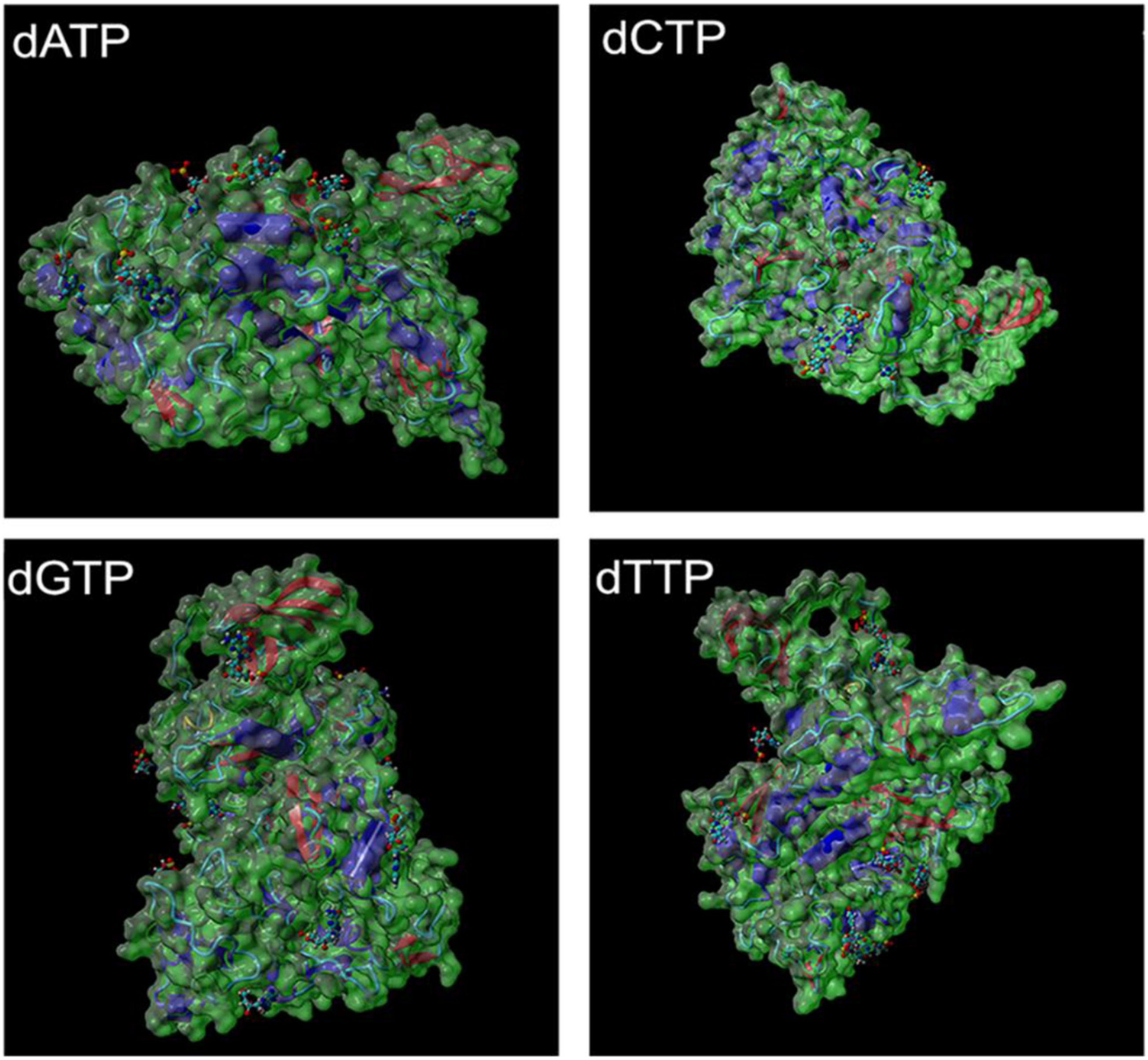

Supplementary Figure 1: Docking analysis of dNTPs with MYH9 a acids 3-778.

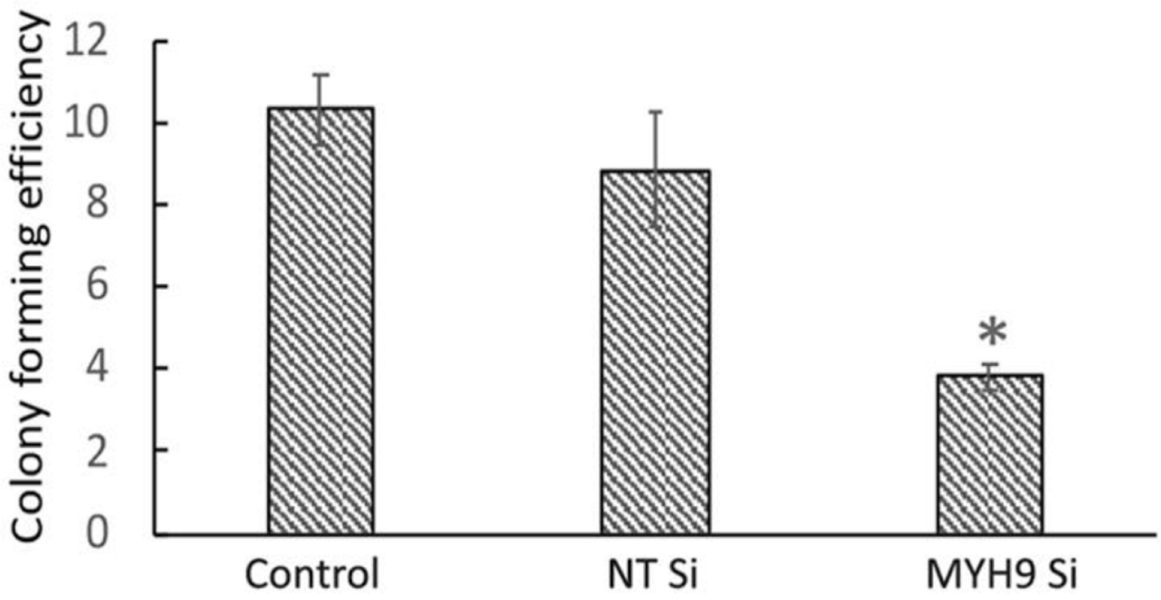

Supplementary Figure 2: Colony forming efficiency in MDA MB 231 cells transfected with NT siRNA and MYH9 siRNA. The numbers are average of 4 readings. Bars represent ± standard deviation. $^{*}p > 0.02$.

Supplementary Table 1: Docking analysis of MYH9 (a acids 3-778) with dNTPs. See Supplementary Table 1

Supplementary Table 2: Oligonuleotides used for PCR detection of dNTPs

Detection primer:

CCGCCTCCACCGCC

dTTP detection template

5′TCGCTCGCTCTTGCCTCGGTCCTCGCTCGCTCTTGCCTCGGTCCTCGCTCGCTCTTGCCTCGGTCCTCGCTCGCTCTTGCCTCGGTC
CTCGCTCGCTCTTGCCTCGGTCCTCGCTCGCTCTTGCCTCGGTCCTCGCTCGCTCTTGCCTCGGTCCTCGCTCGCTCTTGCCTCGGTCC
TTT***A***TTT**GGCGGTGGAGGCGG** 3′

dATP detection template

5′AGACAGACACAAGACACAGACCAGACAGACACAAGAACACAGACCAGACAGACACAAGACACAGACCAGACAGACCAAGACA
CAGACCAGACAGACACAAGACACAGACCAGACAGACACAAGACACAGACCAGACAGACACAAGACACAGACCAGAGAGACACA
ACAGACGGAGG**AAA*T*AAA GGCGGTGGAGGCGG** 3′

dCTP detection template

5′CCACTCACTCTTACCTCAATCCCCACTCACTCTTACCTCAATCCCCACTCACTCTTACCTCAATCCCCACTCACTCTTACCTCAATCC
CCACTCACTCTTACCTCAATCCCCACTCACTCTTACCTCAATCCCCACTCACTCTTACCTCAATCCCCACTCACTCTTACCTCAATCC
TTT*G*TTT GGCGGTGGAGGCGG 3′

dGTP detection template

5′GGAGTGAGTGTGAGGTGAATGATGAGTGAGTGTGAGGTGAATGTAGAGTGAGTGTGAGGTGAATGATGAGTGAGTGTGAGGTGAA
TGTAGAGTGAGTGTGAGGTGAATGATGAGTGAGTGTGAGGTGAATGTAGAGTGAGTGTGAGGTGAATGATGAGTGAGTGTGAGGTGA
ATGG **TTT*C*TTT GGCGGTGGAGGCGG** 3′

The primer binding site is shown as black bold letters at the 3′ end. The Bold italic shows the dNTP detection site.
