# Supplementary material for: MYH9 binds to dNTPs via deoxyribose moiety and plays an important role in DNA synthesis
Source: Oncotarget. 2022 Mar 14;13:534–50. doi: 10.18632/oncotarget.28219 (PMC8923078; doi:10.18632/oncotarget.28219)
Supplement: Supplementary file 2 [file oncotarget-13-28219-s002.docx]

**Supplementary Table 1: Docking analysis of MYH9 (a acids 3-778) with dNTPs. Interacting amino acids**

dTTP

Clu |Bind.energy[kcal/mol]|Dissoc. constant [pM]| Contacting receptor residues

----+---------------------+---------------------+-----------------------------

001 | 000007.7360 | 00000002135191.5000 | ASN 253, GLU 255, ARG 427, VAL 430, LEU 431, ASN 434, LYS 435, ASP 438, LYS 439, THR 440, LYS 441, ARG 442, GLU 610, LEU 611, LYS 613, ASP 614, LYS 656

002 | 000007.7320 | 00000002149655.5000 | PHE 238, ARG 240, ASN 253, ILE 254, GLU 255, THR 256, TYR 257, ARG 424, ARG 427, VAL 430, ASN 434, ARG 442, LEU 611, ASP 614, GLN 653, LYS 656, LEU 657, THR 660, LEU 661

003 | 000007.5540 | 00000002902983.5000 | ILE 110, TYR 111, VAL 120, ASN 122, PRO 123, TYR 124, LYS 125, ASN 126, LEU 127, ILE 129, TYR 130, GLY 177, ALA 178, GLY 179, THR 181, GLU 182, ASN 183, LYS 186

004 | 000007.5470 | 00000002937485.0000 | ASN 253, ILE 254, GLU 255, ARG 424, ARG 427, VAL 430, ASN 434, LYS 435, ASP 438, LYS 439, THR 440, LYS 441, ARG 442, GLU 610, LEU 611, TRP 612, LYS 613, ASP 614, LYS 656

005 | 000007.5220 | 00000003064085.5000 | PHE 238, ARG 240, ASN 253, GLU 255, ARG 424, VAL 430, ASN 434, ASP 438, ARG 442, GLU 610, LEU 611, TRP 612, LYS 613, ASP 614, LYS 656, LEU 657, THR 660

006 | 000007.4550 | 00000003430935.5000 | TYR 113, LEU 116, PHE 117, CYS 118, ARG 143, ILE 153, THR 156, SER 160, ASP 164, GLU 166, GLN 168, ASN 665, PRO 666, ASN 667, PHE 668, ASN 710

007 | 000007.3630 | 00000004007277.7500 | GLU 175, SER 176, GLY 177, ALA 178, GLY 179, LYS 180, THR 181, LYS 185, ASN 215, GLU 219, SER 232, SER 233, ARG 234, PHE 235, LYS 237, ASP 452, ILE 453, ALA 454, GLY 455, GLU 457

008 | 000007.1050 | 00000006193925.0000 | PRO 518, PRO 522, ALA 526, ASP 529, GLU 530, TRP 533, GLN 623, ARG 639, LYS 640, GLY 641, MET 642, PHE 643, ARG 644, GLN 648

009 | 000007.0890 | 00000006363471.5000 | ILE 110, TYR 111, VAL 120, ILE 121, ASN 122, PRO 123, TYR 124, LYS 125, ASN 126, LEU 127, ILE 129, TYR 130, HIS 149, GLY 177, ALA 178, GLY 179, LYS 180, THR 181, GLU 182, ASN 183

010 | 000007.0480 | 00000006819421.0000 | LEU 116, GLU 166, THR 483, MET 484, LEU 487, GLU 488, GLU 491, ASN 665, PRO 666, PRO 709, ASN 710, ARG 711

011 | 000006.8840 | 00000008994170.0000 | ASN 253, ILE 254, GLU 255, THR 256, ARG 424, ARG 427, VAL 430, LEU 431, ASN 434, GLU 610, LEU 611, LYS 613, ASP 614, VAL 615, ASP 616, ARG 617, LYS 656

dGTP

Clu |Bind.energy[kcal/mol]|Dissoc. constant [pM]| Contacting receptor residues

----+---------------------+---------------------+-----------------------------

001 | 000008.3870 | 00000000711618.9375 | ILE 110, TYR 111, VAL 120, ILE 121, ASN 122, PRO 123, TYR 124, LYS 125, TYR 130, GLU 175, SER 176, GLY 177, ALA 178, GLY 179, LYS 180, THR 181, GLU 182, ASN 183, ASP 452, ILE 453, ALA 454, GLY 455, CYS 671

002 | 000007.8710 | 00000001700123.6250 | ARG 240, ASN 253, GLU 255, ARG 424, ARG 427, VAL 430, LEU 431, ASN 434, ASP 438, ARG 442, GLU 610, LEU 611, LYS 613, ASP 614, LYS 656

003 | 000007.8700 | 00000001702995.5000 | LEU 116, PHE 117, ARG 143, ILE 153, THR 156, SER 160, ASP 164, GLU 166, GLN 168, MET 484, ASN 665, PRO 666, ASN 667, PHE 668, PRO 709, ASN 710, ALA 765

004 | 000007.8570 | 00000001740775.0000 | ILE 110, TYR 111, VAL 120, ASN 122, PRO 123, TYR 124, LEU 127, ILE 129, TYR 130, HIS 149, SER 176, GLY 177, GLY 179, LYS 180, THR 181, GLU 182, ASN 183, ILE 453, GLY 455

005 | 000007.8450 | 00000001776391.7500 | SER 176, GLY 177, GLY 179, LYS 180, THR 181, GLU 182, SER 233, ARG 234, PHE 235, GLY 236, LYS 237, ASP 452, ILE 453, ALA 454, GLY 455, GLU 457, ILE 469

006 | 000007.7030 | 00000002257491.5000 | GLU 260, SER 262, ARG 263, ARG 266, GLN 267, ALA 268, GLU 271, PHE 302, SER 304, LEU 461, SER 463, GLU 465, LYS 580, ASP 582, GLU 583, LYS 587

007 | 000007.6620 | 00000002419243.2500 | LEU 116, ARG 143, THR 156, SER 160, GLN 163, ASP 164, GLU 166, GLN 168, ASN 665, ASN 667, PRO 709, ASN 710, ARG 720, ALA 765, GLY 766, LEU 768, ALA 769, GLU 772

008 | 000007.6420 | 00000002502302.0000 | SER 169, PHE 238, ARG 240, ASN 253, ILE 254, GLU 255, VAL 430, LEU 431, ASN 434, ARG 442, PHE 447, GLY 449, GLU 610, LEU 611, LYS 613, ASP 614, LYS 656, LEU 657, THR 660, LEU 661, THR 664

009 | 000007.5000 | 00000003180000.0000 | PRO 518, ALA 519, GLY 520, PRO 522, ALA 526, LEU 527, ASP 529, GLU 530, TRP 533, LYS 545, GLN 548, GLU 549, GLN 623, ARG 639, LYS 640, GLY 641, MET 642, PHE 643, GLN 648

010 | 000007.4110 | 00000003695430.0000 | ASN 122, SER 176, GLY 177, GLY 179, LYS 180, THR 181, GLU 182, LYS 185, GLU 219, SER 232, SER 233, ARG 234, PHE 235, LYS 237, ALA 454, GLY 455, GLU 457, ILE 469

011 | 000007.3940 | 00000003802998.2500 | ILE 110, TYR 111, VAL 120, ASN 122, PRO 123, TYR 124, LYS 125, ASN 126, LEU 127, ILE 129, TYR 130, GLY 177, ALA 178, GLY 179, GLU 182, ASN 183, LYS 186

012 | 000007.2580 | 00000004784267.5000 | PRO 518, PRO 522, ALA 526, ASP 529, GLU 530, TRP 533, PHE 534, GLN 623, VAL 624, ARG 639, LYS 640, GLY 641, PHE 643, GLN 648

013 | 000007.2160 | 00000005135726.5000 | PHE 238, ARG 240, ASN 253, GLU 255, ARG 424, ARG 427, VAL 430, ASN 434, ARG 442, GLU 610, LEU 611, LYS 613, ASP 614, GLN 653, LYS 656, LEU 657, THR 660

014 | 000007.1250 | 00000005988330.5000 | ILE 110, TYR 111, VAL 120, ASN 122, PRO 123, TYR 124, LYS 125, ASN 126, LEU 127, ILE 129, TYR 130, GLY 177, ALA 178, GLY 179, GLU 182, ASN 183, CYS 671

015 | 000006.9830 | 00000007610148.5000 | ALA 22, ASP 25, TRP 26, ALA 28, LYS 29, LYS 30, LEU 31, VAL 32, TRP 33, PRO 43, MET 75, ASN 76, PRO 77, PRO 78, HIS 99

dCTP

Clu |Bind.energy[kcal/mol]|Dissoc. constant [pM]| Contacting receptor residues

----+---------------------+---------------------+-----------------------------

001 | 000007.5230 | 00000003058918.2500 | LEU 116, LYS 142, ARG 143, THR 156, ARG 159, SER 160, GLN 163, ASP 164, GLU 166, GLN 168, ASN 665, ASN 667, PRO 709, ASN 710, ARG 720, LEU 768, ALA 769, GLU 772

002 | 000007.4200 | 00000003639719.2500 | PHE 238, ARG 240, ASN 253, GLU 255, ARG 424, ARG 442, PHE 447, GLY 449, GLU 610, LEU 611, TRP 612, LYS 613, ASP 614, LYS 656, LEU 657, THR 660, LEU 661, THR 664

003 | 000007.3170 | 00000004330797.5000 | TYR 113, LEU 116, PHE 117, CYS 118, ILE 153, THR 156, GLU 166, THR 483, MET 484, GLU 488, ASN 665, PRO 666, ASN 667, PHE 668

004 | 000007.2900 | 00000004532722.5000 | ILE 110, TYR 111, VAL 120, ILE 121, ASN 122, PRO 123, TYR 124, LYS 125, ASN 126, LEU 127, ILE 129, TYR 130, ALA 178, GLY 179, GLU 182, ASN 183, CYS 671

005 | 000007.1990 | 00000005285219.5000 | ILE 110, TYR 111, VAL 120, ILE 121, ASN 122, PRO 123, TYR 124, LYS 125, ASN 126, LEU 127, ILE 129, TYR 130, GLY 177, ALA 178, GLY 179, GLU 182, ASN 183, LYS 186, CYS 671

006 | 000007.1880 | 00000005384261.5000 | PHE 238, ARG 240, ASN 253, ILE 254, GLU 255, THR 256, ARG 427, VAL 430, LEU 431, ASN 434, ASP 438, ARG 442, GLU 610, LEU 611, LYS 613, ASP 614, LYS 656

007 | 000007.1270 | 00000005968150.0000 | PRO 518, ALA 519, PRO 522, ALA 526, LEU 527, ASP 529, GLU 530, TRP 533, LYS 545, GLN 548, GLU 549, GLN 623, ARG 639, LYS 640, GLY 641, MET 642, PHE 643, GLN 648

008 | 000007.0400 | 00000006912125.0000 | ILE 110, VAL 120, ILE 121, ASN 122, PRO 123, TYR 124, LYS 125, TYR 130, SER 176, GLY 177, ALA 178, GLY 179, LYS 180, THR 181, GLU 182, ASN 183, GLY 455, CYS 671

009 | 000006.9050 | 00000008680963.0000 | LEU 116, PHE 117, SER 160, ASP 164, GLU 166, GLN 168, THR 483, MET 484, LEU 487, ASN 665, PRO 666, ASN 667, PHE 668, ASN 710, ARG 711

010 | 000006.8350 | 00000009769640.0000 | ASN 253, ASN 434, LYS 435, ASP 438, LYS 439, ARG 442, GLU 610, LEU 611, TRP 612, LYS 613, ASP 614, LYS 656

011 | 000006.8310 | 00000009835821.0000 | LYS 142, ARG 143, THR 156, ARG 159, SER 160, GLN 163, ASP 164, GLU 166, GLN 168, ASN 665, ASN 667, ASN 710, ARG 711, VAL 712, LEU 768, ALA 769, GLU 772

012 | 000006.6890 | 00000012499654.0000 | ASN 122, PRO 123, TYR 124, LYS 125, SER 176, GLY 177, ALA 178, GLY 179, LYS 180, THR 181, GLU 182, SER 233, PHE 235, GLY 236, LYS 237, ASP 452, ILE 453, ALA 454, GLY 455

dATP

Clu |Bind.energy[kcal/mol]|Dissoc. constant [pM]| Contacting receptor residues

----+---------------------+---------------------+-----------------------------

001 | 000007.5160 | 00000003095273.0000 | TYR 124, GLU 175, SER 176, GLY 177, ALA 178, GLY 179, LYS 180, THR 181, GLU 182, ILE 453, ALA 454, GLY 455, GLU 457, ILE 469, ILE 673, ASN 675, HIS 676, GLU 677, LYS 678

002 | 000007.2560 | 00000004800445.0000 | ILE 110, TYR 111, VAL 120, ASN 122, PRO 123, TYR 124, LYS 125, ASN 126, LEU 127, ILE 129, TYR 130, GLY 177, ALA 178, GLY 179, GLU 182, ASN 183, CYS 671

003 | 000007.2110 | 00000005179250.5000 | PHE 238, ARG 240, ASN 253, ILE 254, GLU 255, ARG 424, ARG 427, VAL 430, ASN 434, ARG 442, PHE 447, GLU 610, LEU 611, LYS 613, ASP 614, LYS 656, LEU 657, THR 660

004 | 000007.0400 | 00000006912125.0000 | TYR 113, GLY 115, LEU 116, PHE 117, CYS 118, ARG 143, ILE 153, THR 156, SER 160, ASP 164, GLU 166, GLN 168, ASN 665, ASN 667, PHE 668, VAL 669, PRO 709, ASN 710, ALA 765, GLY 766

005 | 000006.9840 | 00000007597315.0000 | PRO 518, PRO 522, ALA 526, ASP 529, GLU 530, TRP 533, GLN 623, ARG 639, LYS 640, GLY 641, MET 642, PHE 643, ARG 644, GLN 648

006 | 000006.9760 | 00000007700593.5000 | LEU 116, THR 156, SER 160, ASP 164, GLU 166, GLN 168, THR 483, MET 484, LEU 487, GLU 488, GLU 491, ASN 665, ASN 667, PRO 709, ASN 710, ARG 711

007 | 000006.9470 | 00000008086889.0000 | TYR 113, LEU 116, LYS 142, ARG 143, THR 156, ARG 159, SER 160, GLN 163, ASP 164, GLU 166, GLN 168, ASN 665, ASN 667, ASN 710, GLY 766, LEU 768, ALA 769, GLU 772

008 | 000006.8620 | 00000009334419.0000 | ILE 110, TYR 111, VAL 120, ILE 121, ASN 122, PRO 123, TYR 124, LYS 125, ASN 126, LEU 127, ILE 129, TYR 130, GLY 177, ALA 178, GLY 179, GLU 182, ASN 183, LYS 186, CYS 671

009 | 000006.8320 | 00000009819234.0000 | LEU 116, THR 156, SER 160, ASP 164, GLU 166, GLN 168, THR 483, MET 484, LEU 487, GLU 488, GLU 491, ARG 494, ASN 665, PRO 666, ASN 667, PRO 709, ASN 710, ARG 711

010 | 000006.7780 | 00000010756231.0000 | LEU 116, LYS 142, ARG 143, THR 156, ARG 159, SER 160, GLN 163, ASP 164, GLU 166, GLN 168, ASN 665, PRO 709, ASN 710, ARG 720, GLY 766, LEU 768, ALA 769, GLU 772

011 | 000006.7550 | 00000011181996.0000 | LEU 116, ARG 143, THR 156, SER 160, ASP 164, GLU 166, GLN 168, ASN 665, PRO 666, ASN 667, PRO 709, ASN 710, ARG 764, ALA 765, GLY 766, VAL 767, LEU 768, ALA 769

012 | 000006.6630 | 00000013060393.0000 | PRO 518, ALA 519, GLY 520, PRO 522, ALA 526, LEU 527, ASP 529, GLU 530, TRP 533, LYS 545, GLU 549, GLN 623, ARG 639, LYS 640, GLY 641, MET 642, PHE 643, GLN 648

013 | 000006.6560 | 00000013215613.0000 | PHE 238, ARG 240, ASN 253, ILE 254, GLU 255, THR 256, ARG 427, VAL 430, LEU 431, ASN 434, ARG 442, GLU 610, LEU 611, LYS 613, ASP 614, LYS 656, THR 660

014 | 000006.6560 | 00000013215613.0000 | ASN 253, GLU 255, ARG 427, VAL 430, LEU 431, ASN 434, ASP 438, ARG 442, GLU 610, LEU 611, LYS 613, ASP 614, LYS 656

dRP

Clu |Bind.energy[kcal/mol]|Dissoc. constant [pM]| Contacting receptor residues

----+---------------------+---------------------+-----------------------------

001 | 000003.8090 | 00001614232064.0000 | A ILE 217 A LEU 218 A PHE 221 A MET 326 A ILE 331 A LEU 339 A VAL 342 A MET 425 A PHE 426 A TRP 428 A LEU 429

002 | 000003.6420 | 00002139826688.0000 | A ASN 253 A ILE 254 A GLU 255 A THR 256 A ARG 424 A ARG 427 A VAL 430 A LEU 431 A ASN 434 A LEU 611

003 | 000003.4290 | 00003065559040.0000 | A ALA 214 A ASN 215 A LEU 218 A PHE 235 A LYS 237 A ILE 254 A THR 256 A LEU 429 A VAL 430 A ILE 433

004 | 000003.4200 | 00003112481280.0000 | A GLU 260 A SER 262 A ARG 263 A ARG 266 A SER 463 A ASP 582 A GLU 583 A LYS 587

005 | 000003.3870 | 00003290758912.0000 | A LYS 142 A ARG 143 A ARG 159 A GLN 163 A LEU 768 A ALA 769 A GLU 772

006 | 000003.3650 | 00003415248384.0000 | A GLN 168 A SER 169 A PHE 238 A ARG 240 A PHE 447 A GLY 449 A LYS 656 A LEU 657 A THR 660 A LEU 661 A THR 664

007 | 000003.3500 | 00003502817024.0000 | A ILE 110 A TYR 111 A VAL 120 A PRO 123 A LYS 125 A LEU 127 A ILE 129 A TYR 130 A ASN 183

008 | 000003.1700 | 00004746343936.0000 | A TYR 105 A TYR 106 A GLY 108 A PRO 123 A LYS 125 A LEU 127 A ALA 680 A GLY 681
